# miR-706 inhibits the oxidative stress-induced activation of PKCα/TAOK1 in liver fibrogenesis

**DOI:** 10.1038/srep37509

**Published:** 2016-11-23

**Authors:** Ruili Yin, Duo Guo, Shuxian Zhang, Xiuying Zhang

**Affiliations:** 1 Department of Histology and Embryology, School of Basic Medical Sciences, Capital Medical University, 10 Xi tou tiao, You An Men Wai, 100069, Beijing, China.

## Abstract

Oxidative stress induces the activation of liver fibrogenic cells (myofibroblasts), thus promoting the expression of fibrosis-related genes, leading to hepatic fibrogenesis. MicroRNAs (miRNAs) are a new class of small RNAs ~18–25 nucleotides in length involved in post-transcriptional regulation of gene expression. Wound-healing and remodeling processes in liver fibrosis have been associated with changes in hepatic miRNA expression. However, the role of miR-706 in liver fibrogenesis is currently unknown. In the present study, we show that miR-706 is abundantly expressed in hepatocytes. Moreover, oxidative stress leads to a significant downregulation of miR-706, and the further reintroduction of miR-706 inhibits oxidative stress-induced expression of fibrosis-related markers such as α-SMA. Subsequent studies revealed that miR-706 directly inhibits PKCα and TAOK1 expression *via* binding to the 3′-untranslated region, preventing epithelial mesenchymal transition. *In vivo* studies showed that intravenous injection of miR-706 agomir successfully increases hepatic miR-706 and decreases α-SMA, PKCα, and TAOK1 protein levels in livers of carbon tetrachloride (CCl_4_)-treated mice. In summary, this study reveals a protective role for miR-706 by blocking the oxidative stress-induced activation of PKCα/TAOK1. Our results further identify a major implication for miR-706 in preventing hepatic fibrogenesis and suggest that miR-706 may be a suitable molecular target for anti-fibrosis therapy.

Chronic liver insults, such as viral hepatitis, metabolic or toxic diseases, autoimmune diseases, and non-alcoholic steatohepatitis, lead to the excessive deposition of extracellular matrix (ECM) and to the *destruction of hepatic* cells’ *structure* and function, resulting in the development of liver fibrosis^1^,^2^. The process of liver fibrosis inevitably leads to cirrhosis, pathologically characterized by changes in septa formation and nodules of surviving hepatocytes surrounded with scar tissue. Epidemiological data indicates that cirrhosis kills millions of people worldwide^2^; it is the fourth most common cause of adult death in central Europe and fourteenth in the world^3^.

Due to the lack of effective treatments, liver cirrhosis has become a major health problem. It is necessary to find alternative effective therapeutic strategies that prevent the progression of hepatic fibrosis.

The activation and proliferation of fibroblasts plays a crucial role in hepatic fibrogenesis. Hepatic stellate cells (HSCs) are the major source of activated fibroblasts^4^,^5^. Moreover, recent studies have shown that *activated HSCs*, portal *fibroblasts*, and myofibroblasts of bone-marrow origin have fibrogenic properties^6^,^7^. Further, several *in vitro* and *in vivo* studies have suggested that hepatocytes may assume a fibroblast-like morphology and thus undergo epithelial–mesenchymal transition (EMT)^8^,^9^,^10^. Although clear evidence on hepatocyte EMT in diseased livers is still missing, Zeisberg and colleagues showed that TGF-β treatment induces hepatocyte EMT *in vitro*. Furthermore, microRNAs (miRNAs) are important regulators of EMT through the targeting of different components of signaling pathways. For example, miR-21 activates EMT *via* the PTEN/AKT pathway^11^ and downregulation of miR-101 promotes hepatocyte EMT^12^,^13^.

miRNAs are endogenous small non-coding RNAs that regulate gene expression by binding to the 3′ untranslated region (3′UTR) of the target mRNA^14^,^15^. miRNAs play fundamental physiological roles in a variety of biological processes, including cell proliferation, differentiation, metabolism, immune response, and apoptosis^16^. However, dysregulation of miRNA has been associated with liver diseases, such as hepatic fibrosis, fatty liver disease, viral hepatitis, and hepatocellular carcinoma (HCC)^17^,^18^,^19^. Whereas miR-214 and miR-34c levels are upregulated, miR-122 and miR-29b levels are downregulated during the progression of fibrogenesis^20^,^21^,^22^,^23^. However, the role of miR-706 in liver fibrosis is currently unknown. Interestingly, miR-706 has been related to fatty-acid-regulated insulin resistance in mouse myoblasts^24^, and to vesicular stomatitis virus-induced apoptosis by reducing the activation of caspase-3 and caspase-9^25^. In this study, the levels of miR-706 were found to be significantly downregulated in CCl_4_-induced fibrotic livers. We further investigated the molecular mechanisms of miR-706 in liver fibrosis using bioinformatics indicating that PKCα and TAOK1 might be targeted genes regulated by miR-706.

PKCα plays a crucial role in signal transduction in different organs and tissues^26^. Interestingly, PKCα is activated in cells and tissues undergoing increased oxidative stress-induced disorders^27^ such as ischemic cerebrovascular disease, hematopoietic malignancy, and specifically in chronic liver disease, where PKCα has been shown to promote hepatic fibrogenesis^28^. TAOK1, another potential miR-706-targeted gene, is a MAP kinase kinase kinase (MAP3K) that activates the p38 MAPK^29^,^30^,^31^ cascade exacerbating liver fibrosis *via* overexpression of α-SMA^29^,^30^,^31^,^32^.

In the current study, oxidative-stress-induced hepatocyte EMT was detected *in vivo* using CCl_4_-induced fibrotic liver and *in vitro* using hydrogen peroxide in culture. Here, we show that miR-706 is a novel fibrosis-related liver microRNA, which is hepatocyte specific and nuclear rich, and is downregulated during hepatic fibrogenesis. Our results reveal that miR-706 prevents murine liver fibrosis by attenuating hepatocyte EMT. Furthermore, we characterized *in vivo* the therapeutic potential of miR-706 in hepatic fibrogenesis by using miR-706 agomir injections and *in vitro* by the overexpression of miR-706 in hepatocyte cell lines. In addition, we demonstrated that PKCα and TAOK1 are direct targets of miR-706 in hepatocytes responsible for EMT during liver fibrosis.

## Results

### miR-706 is downregulated in fibrotic livers

**Figure 1:**
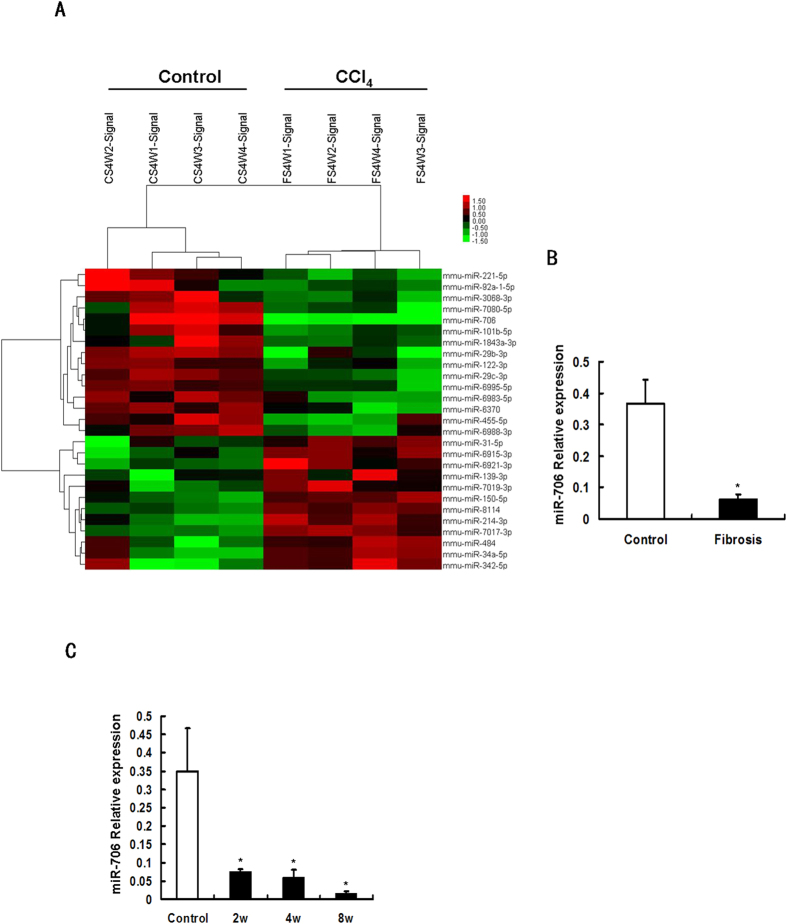
miR-706 is downregulated during liver fibrosis. (**A**) MicroRNA (miRNA) expression was dysregulated in 4-week CCl_4_-treated liver, detected by miRNA microarray. Both down- and upregulated miRNAs were identified in the liver. The expression of miRNAs is hierarchically clustered on the *y* axis, and samples are hierarchically clustered on the *x* axis. The legend on the right indicates miRNAs. Relative expression of miRNAs is depicted according to the color scale shown on the top left. Red indicates upregulation and green indicates downregulation. (**B**) Validation of the miR-706 microarray data by RT-PCR analysis of DNase-I-treated total RNA. RNAs from 4 mice were used for RT-PCR and each sample was analyzed in triplicate. *P < 0.05. (**C**) miR-706 levels were reduced in 2, 4, and 8 weeks of CCl_4_ treatment compared to control tissue and analyzed by real-time PCR, *P < 0.05, n = 4 in each group. Data are expressed as mean ± SEM.

To identify miRNAs involved in hepatic fibrogenesis, we applied a widely-used model of periportal fibrosis using CCl_4_ treatment, characterized by the excessive deposition of ECM, the reconstruction of hepatic lobules and loss of liver function (Supporting Fig. 1A–C). The expression of miRNAs expression in fibrotic liver tissue was analyzed using TLDA, and differentially expressed miRNAs were filtered by the RVM *t* test. A total of 15 miRNAs were significantly downregulated (*P* < 0.05), while 12 miRNAs were significantly upregulated in 4-week CCl_4_-induced fibrotic livers (Fig. 1A). Consistent with previous studies^22^,^23^, the miR-29 and miR-122 families associated with gene encoding for ECM protein and collagen in fibrotic livers were downregulated in our analysis. Interestingly, we observed that miR-706, a miRNA not yet reported in any liver disorder, was dramatically downregulated in CCl_4_-induced fibrotic livers. Our results were further validated by the levels of mRNA transcripts for miR-706 using qPCR (Fig. 1B). Moreover, miR-706 progressively decreased throughout the 8-week period of CCl_4_ treatment (Fig. 1C).

### miR-706 is abundant in normal hepatocytes and downregulated in fibrotic hepatocytes

**Figure 2:**
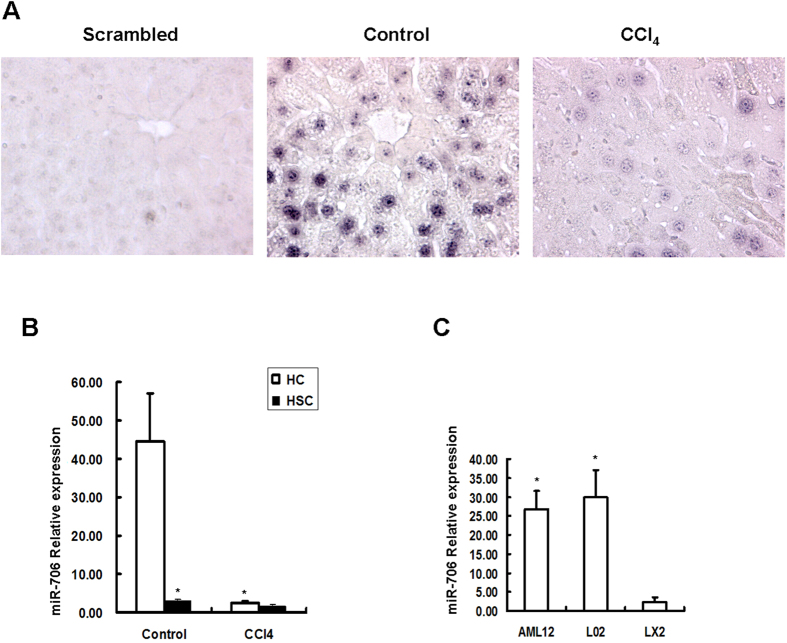
miR-706 is expressed mainly in hepatocytes, but not in HSCs, and its expression decreases in fibrotic liver. (**A**) Localization of miR-706 in livers using ISH. Tissue sections were hybridized to biotin-labeled oligos (Scrambled oligo probe and anti-miR-706), captured with alkaline phosphatase-conjugated streptavidin, and the signal (blue dot) was developed with nitro blue tetrazolium/5-bromo-4-chloro-3-indolyl phosphate (NBT/BCIP), n = 4 in each group. (**B**) miR-706 expression in isolated hepatocytes and HSCs from CCl_4_-treated and control livers was analyzed by RT-PCR, *P < 0.05, n = 4 in each group, HC: hepatocytes, HSC: hepatic stellate cells. (**C**) miR-706 expression was abundant in murine and human hepatocyte cell lines AML12 and L02, respectively, but negligible in human stellate LX2 cells, *P < 0.05.

Previous studies have shown that hepatocytes and HSCs are the main intrinsic cell sources which contribute to the development of liver fibrosis^4^,^5^,^8^. To further clarify the biological roles of miR-706 in hepatic fibrosis, we performed *in situ* hybridization (ISH). Here, we aimed to detect the levels and the location of miR-706 in both CCl_4_-induced fibrotic livers and olive-oil control livers. We found that miR-706 expression levels in hepatocytes of fibrotic livers was dramatically lower than that of control livers. However, little expression of miR-706 was detected in nonparenchymal cells in both fibrotic and in control livers. Additionally, ISH using scrambled RNA did not produce any signal (Fig. 2A). Interestingly, miR-706 expression was scarce in human fibrotic liver and bile duct ligation (BDL)-induced fibrotic liver (Supporting Fig. 2A,C). Furthermore, the transcript levels of miR-706 confirmed its expression in isolated hepatocytes from normal control livers, whereas miR-706 expression was significantly decreased in isolated hepatocytes from fibrotic livers. Noticeably, the transcript levels of miR-706 were significantly lower in isolated HSCs from both control and fibrotic livers (Fig. 2B, Supporting Fig. 2B). Similarly, miR-706 was substantially expressed in immortalized mouse and human hepatocyte AML12 and L02 cell lines, but barely detectable in the human stellate cell line LX2 (Fig. 2C). These results indicated that miR-706 was abundant in normal hepatocytes but not in HSCs.

### Oxidative stress-induced hepatocyte EMT is associated with decreased levels of miR-706

**Figure 3:**
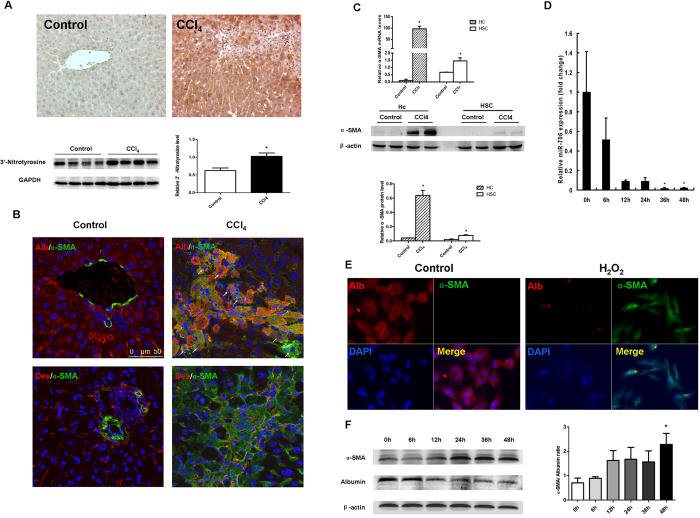
Increased oxidative stress and hepatocyte EMT is characteristic of 4-week CCl_4_-treated liver. *In vitro* treatment with H_2_O_2_ induced human hepatocyte cell line L02 EMT, associated with decreased miR-706. (**A**) Protein levels of 3′-nitrotyrosine in CCl_4_-treated and control livers were examined by immunohistochemistry and Western blot, *P < 0.05, n = 4 in each group. (**B**) Representative dual-immunofluorescence staining of albumin and α-SMA, and desmin and α-SMA. (**C**) mRNA and protein expression for α-SMA in hepatocytes and HSCs isolated from CCl_4_-treated and control livers. For real-time PCR, GAPDH was used as an internal control. Hc: hepatocytes, *P < 0.05, n = 4 in each group. Data are expressed as mean ± SEM. (**D,F**) Human hepatocyte cell line L02 was treated with 300 μM H_2_O_2_ for 0, 6, 12, 24, 36 and 48 h, respectively. miR-706 expression was examined by RT-PCR (**D**), *P < 0.05. (**E**) Protein levels of albumin and α-SMA expression was determined by dual-immunofluorescence staining in 2-day H_2_O_2_-treated and control L02 cells, and (**F**) by Western blot. β–actin was used as loading control.

In the liver, CCl_4_ is metabolized by the cytochrome p450 isoenzyme 2E1, generating reactive oxygen species (ROS), which increase ECM deposition and induce the development of hepatic fibrosis^33^. In the current study, we detected increased levels of ROS characterized by 3′-nitrotyrosine-positive hepatocytes in fibrotic livers, compared to control livers (Fig. 3A). Additionally, hepatocytes gained the expression of mesenchymal markers such as α-SMA; however, some α-SMA-positive cells were also positive for desmin, very likely indicating that hepatocyte in partly contributed to the source of pro-fibrogenic fibroblasts (Fig. 3B). These results were further confirmed by the mRNA and protein expression of α-SMA detected in hepatocytes isolated from CCl_4_-treated livers but not from control livers (Fig. 3C). To ensure the lack of cross-contamination, we stained cells used in Fig. 3C with HNF-4, a hepatocyte (HCs) marker, which was not detected in HSCs from either control or CCl4-treated livers (Supplemental Figure 2B). Similarly, desmin, an HSCs marker, was not detected in HCs from either control or CCl4-treated livers (Supplemental Figure 2B). These data suggested that hepatocytes undergo EMT during liver fibrogenesis.

In order to determine the mechanisms of ROS-induced hepatocyte EMT, we administered 300 μM H_2_O_2_ to L02 cells for 2 days and collected cells from 6 to 48 h thereafter. The addition of H_2_O_2_ markedly decreased the levels of miR-706 in L02 cells in a time-dependent manner (Fig. 3D) and induced a fibroblast-like phenotype characterized by increased α-SMA expression and decreased albumin expression as assessed by immunofluorescence (Fig. 3E). To further confirm our results, we analyzed protein expression by Western blot. The overexpression of α-SMA and downregulation of albumin protein levels and increased α-SMA/albumin ratio (Fig. 3F) indicated a shift towards a mesenchymal phenotype. Consistent with our results in the human L02 cell line, H_2_O_2_ stimulation induced EMT in the AML12 murine hepatocyte cell line and primary hepatocytes, a mechanism associated with the downregulation of miR-706 (Supporting Fig. 3).

### miR-706 inhibits oxidative stress-induced PKCα and TAOK1 signaling cascade

**Figure 4:**
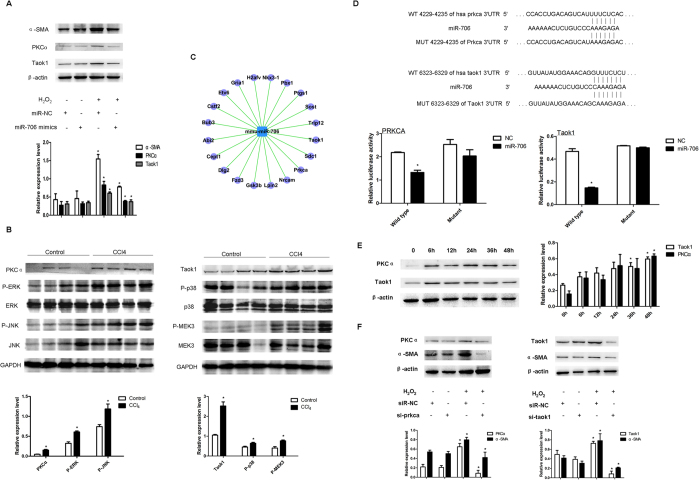
miR-706 inhibits oxidative stress-induced fibrotic related genes in L02 cells. (**A**) Introduction of miR-706 represses H_2_O_2_-stimulated expression of α-SMA, PKCα and TAOK1. L02 cells were transfected with negative control (NC) or miR-706 duplex for 8 h, and then stimulated with 300 μM H_2_O_2_ or remained untreated for 48 h before immunoblotting analysis for α-SMA, PKCα, and TAOK1. β-actin was used as an internal control for immunoblotting. *P < 0.05. (**B**) Upregulation of PKCα and TAOK1 signaling cascade in 2-week CCl_4_-fibrotic murine livers. ERK, JNK, TAOK1, p38, and MEK3 were analyzed by Western blot. GAPDH was used as an internal control. *P < 0.05. (**C**) The miR-706 gene network predicted in 4-week CCl_4_-treated liver compares to controls. In network, blue box node represented miR-706, and purple nodes represented target mRNAs. Edges describe inhibitive effects of miR-706 on mRNAs. (**D**) miR-706 and its putative binding sequences in the 3′UTR of PKCα and TAOK1. miR-706 sequence and the wild-type and mutant 3′UTR segment of PKCα and TAOK1 are shown. Mutations were generated in the complementary site that binds to the seed region of miR-706. Expression of miR-706 inhibits the activity of the luciferase reporter containing the wild-type 3′UTR of PKCα and TAOK1. 293 T cells were co-transfected with NC or miR-706 duplexes and psiCHECKTM-2 Vectors carrying either the wild-type (WT) or the mutant (MUT) 3′UTR of PKCα and TAOK1. * *P* < 0.05. (**E**) H_2_O_2_ stimulation induced PKCα and TAOK1 upregulation. L02 cells were treated with 300 μM H_2_O_2_ for 0, 6, 12, 24, 36, and 48 h, respectively. PKCα and TAOK1 expression was examined by Western blot. β–actin was used as loading control. *P < 0.05. (**F**) Inhibition of PKCα and TAOK1 attenuated the H_2_O_2_-induced expression of α-SMA. L02 cells were transfected with the indicated duplex of human siPrkca or siTaok1 for 8 h, then stimulated with 300 uM H_2_O_2_ (+) or remained untreated (−) for 48 h before immunoblotting. β–actin was used as an internal control. *P < 0.05.

Since our results showed that H_2_O_2_ induced EMT and that this phenomenon was associated with decreased levels of miR-706, we used miR-706 mimics. The reintroduction of miR-706 significantly inhibited the ROS-induced EMT (Fig. 4A), further indicating that miR-706 downregulation may facilitate oxidative stress-induced hepatocyte mesenchymal transition. Similar findings were also reproducible using the murine AML12 cell line (Supporting Fig. 4A).

Next, we investigated the molecular mechanisms by which miR-706 attenuates hepatic fibrogenesis. Increase in ROS activates PKCα, a well-known promoter of hepatic fibrogenesis *via* phosphorylation of MAPK, including ERK and JNK^33^,^34^,^35^. As shown in Fig. 4B, increased PKCα protein levels were detected in 2-week CCl_4_-induced fibrotic livers, associated with higher phosphorylation of ERK and JNK. TAOK1 is a MAP3K that activates p38 MAPK and MEK3/6^31^. Thus, we next validated that increase in TAOK1 protein levels correlated with higher phosphorylation of p38 MAPK and MEK3 in 2-week CCl_4_-induced fibrotic livers, (Fig. 4B). Interestingly, our bioinformatic analysis predicted that PKCα and TAOK1 were potentially regulated by miR-706 (Fig. 4C), and the putative miR-706 binding sites in the 3′-untranslated region (UTR) of PKCα and TAOK1 were predicted, as shown in Fig. 4D. Moreover, dual-luciferase reporter analysis indicated that co-expression of miR-706 significantly inhibited firefly luciferase expression with wild-type but not using the mutant 3′UTR of PKCα or TAOK1 (Fig. 4D). *In vitro* studies further confirmed that treatment with H_2_O_2_ resulted in increased PKCα and TAOK1 expression in L02 hepatocytes (Fig. 4E), and that the knockdown of PKCα and TAOK1 attenuated H_2_O_2_-induced α-SMA expression (Fig. 4F). The restoration of miR-706 attenuated H_2_O_2_-induced PKCα and TAOK1 protein levels, consequently inhibiting the increase in α-SMA (Fig. 4A). Consistently, miR-706 inhibition significantly increased the expression of PKCα and TAOK1 levels (Supporting Fig. 4B). All together, these results suggest that miR-706 may directly suppress PKCα and TAOK1 translation by binding to the 3′UTR and thus attenuate the oxidative stress-induced hepatocyte EMT.

### Restoration of miR-706 expression alleviates hepatic fibrogenesis *in vivo*

**Figure 5:**
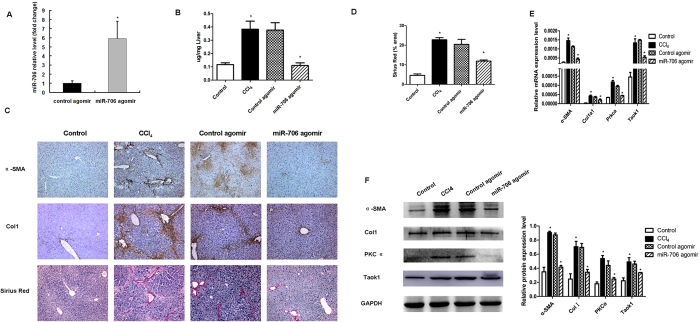
Gene transfer of miR-706 prevents CCl_4_-induced liver fibrosis in mice. (**A**) Administration of miR-706 agomir enhanced miR-706 levels in livers analyzed by RT-PCR after 1 week of the second injection of miR-706 agomir, *P < 0.05. (**B**) Quantification of hepatic hydroxyproline content; the data are expressed as hydroxyproline (μg)/liver wet weight (mg). *P < 0.05. (**C**) Decreased α-SMA, Col1a1 expression, and Sirius Red staining were detected by immunohistochemistry staining in 6-week CCl_4_-treated livers injected with miR-706 agomir. (**D**) Quantification of the Sirius red-positive area. *P < 0.05. (**E**) mRNA expression of α-SMA, col1a1, prkca and Taok1 were detected by real-time PCR. GAPDH was used as an internal control. *P < 0.05. (**F**) Decreased α-SMA and Col1 expression and inhibited PKCα and TAOK1 were detected by Western blot in 6-week CCl_4_-treated livers injected with miR-706 agomir. The levels of target genes in each sample were normalized to that of GAPDH (internal control). *P < 0.05.

We next investigated whether reintroduction of miR-706 could attenuate CCl_4_-induced hepatic fibrosis *in vivo*. After 4 weeks of CCl_4_ treatment, mice were injected intravenously either with control agomir or miR-706 agomir once per week for a period of 2 weeks. Injection of miR-706 agomir increased miR-706 levels in liver tissue (Fig. 5A). Quantitative analysis revealed that miR-706 significantly decreased hydroxyproline content compared to control agomir (Fig. 5B), and greatly attenuated the severity of liver fibrosis, as demonstrated by α-SMA, Col1a1, Sirius red staining (Fig. 5C,D). Moreover, Western blot analysis indicated that treatment with miR-706 agomir markedly inhibited α-SMA, Col1a1, PKCα, and TAOK1 expression in livers of CCl_4_-treated mice compared with those injected with control agomir (Fig. 5F). These data indicated that miR-706 may attenuate liver fibrosis progression to some extent. Furthermore, treatment with miR-706 agomir significantly reduced the mRNA levels of α-SMA, Col1a1, Prkca, and Taok1 in the livers of CCl_4_-treated mice (Fig. 5E). Furthermore, the injection of miR-706 antagomir promoted liver fibrosis characterized by increased α-SMA and Col1 expression, associated with high PKCα and TAOK1 expression (Supporting Fig. 5). All together, these results indicated that miR-706 may attenuate hepatic fibrogenesis *in vivo via* downregulation of PKCα and TAOK1 expression.

### miR-706 has little effect on oxidative stress-induced apoptosis in hepatocytes

**Figure 6:**
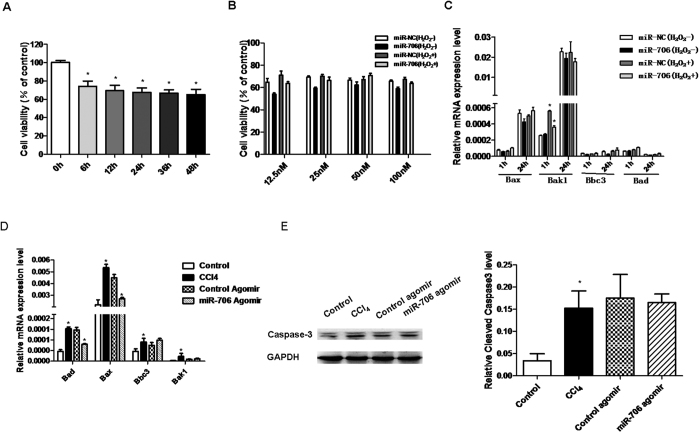
miR-706 has a minor effect on oxidative stress-induced apoptosis in hepatocytes. H_2_O_2_ (300 uM) induced decreased cell viability measured with CCK8 in hepatic L02 cells, *P < 0.05. n = 4. (**B**) Introduction of miR-706 has little effect on cell viability in hepatic L02 cells. L02 cells were transfected with negative control (NC) or miR-706 (at a concentration of 12.5 nM, 25 nM, 50 nM and 100 nM, respectively) duplex for 8 h, and then stimulated with 300 μM H_2_O_2_ or remained untreated for 6 h before measurement with CCK8, n = 4. (**C**) Hepatic L02 cells were transfected with negative control (NC) or miR-706 duplex for 8 h, and then stimulated with 300 μM H_2_O_2_ or remained untreated for 1 h and 24 h, and pro-apoptotic genes Bax, Bak1, Bbc3, and Bad were measured by real-time PCR. GAPDH was used as an internal control. * *P* < 0.05. (**D**) mRNA expression of Bax, Bak1, Bbc3, and Bad were detected by real-time PCR and (**E**) cleaved caspase-3 expression was detected by Western blot in 6-week CCl_4_-treated livers injected with miR-706 agomir. GAPDH was used as an internal control. *P < 0.05.

Previous studies have described oxidative stress-induced apoptosis of L02 cells^36^. In the current study, decreased cell viability was detected by H_2_O_2_ treatment from 6 h to 48 h (Fig. 6A), however, a certain proportion of cells still survived. These results suggest that surviving cells might undergo EMT. Then we investigated the effects of miR-706 on hepatocyte apoptosis. We found that miR-706 had little effect on H_2_O_2_-induced decreased hepatocyte cell viability and pro-apoptotic genes Bax, Bak1, Bbc3, and Bad (Fig. 6B,C). In our *in vivo* study, we found that CCl_4_ treatment induced increased expression of the pro-apoptotic genes Bax, Bak1, Bbc3, and Bad. Among them, Bax and Bad can be inhibited by adding miR-706 agomir. However, miR-706 agomir did not prevent the increase in expression of Bak1 and Bbc3 induced by CCl_4_ treatment (Fig. 6D). Furthermore, CCl_4_ treatment induced increased cleaved Caspase-3 expression, which was not alleviated by miR-706 agomir injection (Fig. 6E). These results indicate that miR-706 may inhibit some pro-apoptotic genes during liver fibrosis, but have no direct effect on overall hepatocyte apoptosis. Further mechanisms need to be investigated in future studies.

## Discussion

The progression of liver fibrosis leads to irreversible cirrhosis, end-stage hepatocellular carcinoma and, ultimately, to liver failure. Whereas liver fibrosis is an asymptomatic disorder, the progression from fibrosis to liver cirrhosis considerably increases the risk of mortality and morbidity^37^. Thus, for the prevention of hepatic fibrogenesis, it is essential to find an effective treatment. Activated fibroblasts are characterized by their contractile capacity, the deposition of ECM and subsequently the production of hepatic fibrosis^38^. HSCs are regarded as the main source of fibroblasts^4^. Moreover, profibrogenic fibroblasts can also originate from portal fibroblasts, bone marrow stem cells and hepatocytes undergoing EMT^6^,^7^,^8^. Recent reports have shown that hepatocyte EMT contributes to the development of liver cirrhosis in CCl_4_-treated rats^10^. In our study, we detected that hepatocytes acquire typical markers of profibrogenic fibroblasts not only in CCl_4_-induced fibrosis, but also in a H_2_O_2_-treated hepatocyte cell line. Hepatocytes undergoing EMT lose the expression of hepatocyte markers such as albumin but gain mesenchymal marker (α-SMA). Some reports indicated that the activation of EMT can be enhanced by the MAPK pathway, regulated by PKCα^41^,^44^. In the present study, increased PKCα and phosphorylation of MAPK JNK and ERK were characteristic of CCl_4_-treated fibrotic livers, indicating that PKCα signal cascade promotes hepatic fibrogenesis.

Oxidative stress is a key factor in the activation of PKCα. In experiments using long-term administration of CCl_4_, increased oxidative stress causes the oxidation of diacylglycerol (DAG), an agonist of PKCα, and the translocation of PKCα from the cytosol to the membrane where it becomes active^41^,^42^,^43^,^44^. In the present study, it increased the expression of 3′-nitrotyrosine, an indicator of ROS, which was also detected in fibrotic liver tissue.

MiRNAs play a crucial role in the regulation of gene expression during hepatic fibrogenesis. In fact, miR-21 promotes hepatic fibrosis *via* the PTEN/AKT pathway^44^ and miR-33a activates HSCs through the PI3K/AKT pathway^21^. Moreover, miR-122 is the most abundant and liver-specific miRNA in the adult human liver, where its overexpression represses collagen production and HSCs activation^22^. The miR-29 family is clearly involved in liver fibrosis, since the downregulation of miR-29 contributes to the HSCs activation through the NF-κB pathway^23^,^46^,^47^. Consequently, the aberrant expression of miRNAs may lead to hepatic fibrogenesis through different mechanisms. In contrast to previous studies, we provided new evidence for the functional role of miR-706 in both murine and human liver fibrosis. Our miRNA microarray analysis indicated that miR-706 was significantly decreased in fibrotic liver. These results were further validated by RT-PCR. By ISH, we determined that the expression of miR-706 was located in the nuclei of normal hepatocytes. Interestingly, the expression of miR-706 was barely detectable in fibrotic liver and non-parenchymal cells. Altogether, these results indicated that miR-706 is a hepatocyte-specific and nucleus-rich miRNA. Concomitant with our data, miR-706 was reported as a nuclear-enriched miRNA^48^. Since no data were available on the function of human miR-706, we next sought to locate the expression of miR-706 in human hepatocytes. MiR-706 expression was detected by ISH in human liver adjacent to carcinoma tissue. Additionally, we confirmed our results using the human hepatocyte cell line L02, the murine hepatocyte cell line AML12, and the human hepatic stellate cell line LX-2. We found decreased miR-706 expression in H_2_O_2_-treated L02 and AML12 cells, further confirming that miR-706 is hepatocyte specific and suggesting that its downregulation can lead to increased oxidative stress. However, the definite molecular mechanism by which miR-706 regulates liver fibrosis has yet to be investigated.

Interestingly, bioinformatics analysis predicted PKCα and TAOK1 as targets of miR-706. Indeed, both PKCα and TAOK1 are important mediators of the MAPK signaling cascade. Thus, we constructed pMIR-report plasmids which encode a firefly luciferase transcript with either a wild-type or a mutant 3′-UTR of human PKCα and TAOK1. We then assessed their respective dual luciferase reporter activities after co-transfection with miR-706 mimics or negative control in 293-T cells. From these experiments, we conclude that miR-706 can directly downregulate the expression of PKCα and TAOK1.

The MAPK signaling pathway and the process of EMT are major contributors to hepatic fibrogenesis. In the current study, increased levels of PKCα and TAOK1 were observed in CCl_4_-induced fibrotic livers associated with increased phosphorylation of JNK, ERK, p38, and MEK3, important mediators of the MAPK pathway. Similarly, increased PKCα and TAOK1 was detected in H_2_O_2_-treated hepatocytes. To further determine the regulation of PKCα and TAOK1 in hepatic EMT, suppression of PKCα and TAOK1 by siRNA inhibited H_2_O_2_-induced α-SMA expression in the human hepatocyte cell line L02.

To further confirm that dysregulation of miR-706 promotes fibroblast-like transformation of hepatocytes, we treated hepatocytes with miR-706 mimics. Interestingly, the overexpression of miR-706 reduced the H_2_O_2_-induced expression of PKCα and TAOK1, consequently blocking α-SMA expression. Concomitantly, miR-706 inhibition promoted H_2_O_2_-induced expression of PKCα and TAOK1. These findings implied that miR-706 reduces hepatic fibrosis by targeting PKCα and TAOK1-dependent regulation of the MAPK cascade.

To further investigate the *in vivo* effects of miR-706 on hepatic fibrogenesis, the reintroduction of miR-706 was established after 4 weeks of CCl_4_ treatment. The restoration of miR-706 dramatically reduced the amount of α-SMA associated with lower levels of PKCα and TAOK1. These results indicated that miR-706 may attenuate the progression of liver fibrogenesis, particularly at early time-points. Furthermore, the inhibition of miR-706 using antagomir aggravated hepatic fibrosis. Altogether, these results revealed that the remission of liver fibrosis can be attributed to the anti-fibrotic role of miR-706 *via* reversing EMT in hepatocytes. Therefore, the regulation of miR-706 expression may be a potentially useful therapeutic approach for attenuating the progression of liver fibrosis.

## Materials and Methods

### Animal models of liver fibrosis

C57BL/6 male mice were purchased from Beijing Vital River Laboratory Animal Technology Co., Ltd. (Beijing, China), bred in our facility, and used as controls or CCl_4_-treated groups. Only eight-week-old mice were used in this study. To induce liver fibrosis, mice were intraperitoneally injected with CCl_4_ (1:9 in olive oil) at a dose of 0.1 mL/kg body weight, twice weekly. Control animals were injected exclusively with olive oil. Animals were sacrificed under anesthesia at 2, 4, 6 and 8 weeks after CCl_4_ treatment. Part of the liver tissue was fixed using 10% buffered formalin overnight before embedding in paraffin or snapped frozen in liquid nitrogen and stored at −80 °C. Part of the liver tissue was stored in RNAlater solution or kept at −80 °C for western blot analysis. Serum was collected and analyzed for alanine aminotransferase (ALT) activity. All animal experiments were conducted in accordance with the Guide for the Care and Use of Laboratory Animals and approved by the Animal Experimentation Ethics Committee of the Capital Medical University.

### MicroRNA array

RNA was isolated from liver tissue using Trizol (Invitrogen, USA) and processed using a miRNA microarray according to the manufacturer’s protocol. TaqMan Low Density Array (TLDA, Invitrogen, USA) on 7900HT Fast Real-Time PCR system (ABI, USA) was applied and the random variance model (RVM) *t* test was used to filter the differently expressed miRNAs. After significant and false discovery rate (FDR) analysis, differentially expressed miRNAs were selected according to their *P* value thresholds^49^.

### Reintroduction of miR-706 in the liver

CCl_4_-treated mice were randomly divided into four groups after 4 weeks; miR-706 agomir negative control, miR-706 agomir, miR-706 antagomir negative control and miR-706 antagomir. All mice from all groups received tail vein injections of miRNA agomir/antagomir or their respective controls at a dose of 2 nmol for 2 weeks, once per week. miRNA agomir/antagomir and their respective controls were purchased from Biolino (Beijing, China). The agomir sequences are shown in Table 1.

### Histological and immunohistochemical analyses

Formalin-fixed liver tissues were embedded in paraffin and cut into 4-μm sections. Sections were dewaxed in xylene and rehydrated in a series of descending percentages of ethanol. Liver specimens were incubated with primary antibody against α-SMA (Sigma-Aldrich), Col1 (Abcam), 3′-nitrotyrosine (Life). An UltraSensitive^TM^ S-P kit (MXB, China) and DAB kit (Zhongshanjinqiao, China) were used. For histopathological examination, H&E, Masson’s trichrome, and Sirius Red staining were used to assess the extent of fibrosis.

### Immunofluorescence staining

Cells were grown on chamber slides and fixed with 4% paraformaldehyde in PBS for 10 min then permeabilized with 0.3% Triton X-100 in PBS for 10 min. Frozen tissues were cut into 4-μm sections, fixed in acetone for 10 min at −20 °C, then dried in air for 1 h. After treatment with blocking solution (1% donkey serum in PBS) for 1 h at room temperature, slides were incubated with rabbit anti-albumin (Proteintech) antibody (1:100) or rabbit anti-E-cadherin (Cell Signaling technology) antibody (1:100) and mouse anti-α-SMA (Sigma-Aldrich) antibody (1:2000) overnight at 4 °C, followed by donkey anti-fluorescein isothiocyanate-labeled anti-mouse IgG (1:200) and Cy3-labeled donkey anti-rabbit IgG (1:200) antibodies for 30 min at room temperature. Nuclear counterstain was performed by 4′,6-diamidino-2-phenylindole (DAPI, Invitrogen). Tissue sections were analyzed by fluorescence microscopy.

### Western blot analysis

Protein from frozen liver tissue (30 mg) or from cells was homogenized and then centrifuged at 12,000 rpm for 15 min, then separated in a 10% sodium dodecyl sulfate (SDS) polyacrylamide gel electrophoresis (PAGE) and transferred to polyvinylidene fluoride (PVDF) membranes (Merck Millipore, Billerica, MA, USA) using a transblot system (Bio-Rad; Hercules, CA, USA). The membranes were blocked with 5% non-fat milk in TBS-T for 1 h at room temperature and then incubated with specific antibodies against PKCα, TAOK1, 3′-Nitrotyrosine, cleaved Caspase-3, α-SMA, Col1a1, total MEK3, pMEK3 (S189), p38 MAPK, p-p38 MAP Kinase (Thr180/Tyr182), JNK, p-JNK, ERK, p-ERK, β–actin and GAPDH. After washing, the PVDF membranes were incubated with horseradish peroxidase-conjugated secondary antibodies. Protein bands were visualized by chemiluminescence (Bio-Rad). The intensity of each band was quantified by densitometric analysis.

### Dual luciferase reporter assay system

The potential miR-706 binding targets were predicted using TargetScan (www.targetscan.org) and miRBase (microRNA.sanger.ac.uk) databases. The sequence of segments with wild-type or mutant seed regions of PKCα and TAOK1 were synthesized and cloned into psiCHECKTM-2 Vectors (Promega, Madison, WI) between NotI and XhoI restriction sites. The synthesized oligos are shown in Table 1. All constructs were verified by sequencing. The 293-T cells (1 × 10^4^ cells/well) transiently transfected with miR-706 (50 nM) or miR-control (50 nM) were seeded in 96-well plates. psiCHECKTM-2 Vectors (50 ng/well) were co-transfected using lipofectamine 2000 (Invitrogen). Cells were harvested 48 h post-transfection and luciferase activities were analyzed by the dual-luciferase reporter assay system (Promega, Madison, WI).

### Cell transfection

miR-706 mimic and scrambled oligonucleotides were purchased from Oligobio (China) and transfected into L-02 and AML12 cell lines (ATCC, Manassas, VA) using Lipofectamine 2000 reagent (Invitrogen, USA) according to the manufacturer’s instructions. siRNA oligonucleotides (Oligobio, China) were used to target the PKCα and TAOK1 transcripts (Table 1). After 8 h of transfection, cells were treated with 300 μM H_2_O_2_ for 1 h and 24 h harvested for mRNA analysis of bax, bad, bak, and bbc3, or for 48 h and harvested for protein analysis of α-SMA, PKCα, and TAOK1.

### RT-PCR analysis

Total RNA was isolated from cells or liver tissues stored in RNAlater solution (Ambion, USA), using Trizol (Invitrogen) and treated with DNase I (Invitrogen) according to the manufacturers’ protocols. Quantitative real-time PCR of miR-706, α-SMA, col1a1, taok1, prkca, bax, bad, bak, and bbc3 was performed using SYBR Green Master mix on a 7500 system (Applied Biosystems, Foster City, CA). Standardization was performed using U6 primers for miR-706, and GAPDH for the other primers, and evaluated using the comparative CT Method (2^−ΔΔCT^). Semi-quantitative RT-PCR of HNF-4 and desmin were performed using hot start Taq Master Mix (Biolab, UK) according to manufacturer’s instructions. The primers are shown in Table 1.

### *In situ* hybridization

For *in situ* hybridization (ISH), 10% buffered formalin-fixed liver tissues were embedded in paraffin and sliced into 4 μm sections, then deparaffinized in xylene, rehydrated in degraded ethanol, and finally in distilled water. The ISH was performed according to the manufacturer’s protocol (Boster, China). Briefly, tissue sections were treated with 3% H_2_O_2_ at room temperature for 10 min, pre-hybridised in the Exiqon hybridization buffer at 38 °C for 4 h, then hybridised with 20 μL miR-706 probe at 38 °C overnight, washed with 2 × SSC, 0.5 × SSC, and 0.2 × SSC buffers for 10 min at 38 °C, respectively. Following the blocking step in the blocked Exiqon at 37 °C for 30 min, sections were treated with alkaline phosphatase-conjugated mouse anti-digoxigenin (diluted 1:200 in 0.5 M TBS) at 37 °C for 60 min, and then treated with developing reagent for 30 min. For human tissue, commercially available adult human liver tissue adjacent to areas of HCC and fibrotic liver tissue arrays were obtained from Shanghai Zhuoli Biotechnology Co., Ltd (Zhuoli Biotechnology Co, Shanghai, China) with the approval of the ethics committee of Capital Medical University. Tissue arrays contained 20 points of a 1.5 mm diameter disk of formalin-fixed, paraffin-embedded tissues representing 8 cases of liver tissue adjacent to areas of HCC and 12 cases of fibrotic liver from individuals aged 25–75 years.

### Isolation of mouse hepatocytes and HSCs

Hepatic cells were isolated using a well-known protocol^50^,^51^. Briefly, mice were anesthetized with pentobarbital sodium (40 mg/kg) intraperitoneally. After the use of a calcium and magnesium-free Hanks’ balanced salt solution containing 0.5 mM EDTA (Invitrogen), livers were perfused *in situ* through the portal vein with Dulbecco’s modified Eagle’s media (DMEM; Invitrogen) containing collagenase (Sigma), until the liver became soft. Then the liver was removed and gently shaken in DMEM containing collagenase at 37 °C for 10 min. The homogenate was filtered and centrifuged at 50 g for 1 min, the pellet washed three times with DMEM in order to harvest hepatocytes, and the supernatant was removed and placed into a new 15-mL centrifuge, using a density-gradient-based separation for culture of HSCs.

### Determination of Liver Hydroxyproline Content

Briefly, wet liver samples (100 mg) stored at −80 °C were homogenized in 1 mL 6 mol/L HCl and incubated for 5 h at 95 °C, then the amount of hydroxyproline was assessed according to the procedure outlined in the Hydroxyproline Testing Kit (A030-2, Jiancheng, Nanjing, China). The optical density of the reaction product was read at 550 nm using a spectrophotometer and expressed as μg/mg wet tissue.

### Viability detection by CCK8

L02 cells were treated with H_2_O_2_ (300 μM) for 6 to 48 h, and then 10 μL CCK8 (Dojindo, Kumamoto, Japan) was added to the cells, and the viability of the cells was measured at 490 nm using an ELISA reader (BioTek, Winooski, VT, USA) according to the manufacturer’s instructions.

### Statistical Analysis

Data are shown as mean ± standard error (SEM), and were obtained in at least three independent experiments. Student’s *t* test was used to analyze the differences between the two groups. ANOVA was performed when more than two experimental groups were used.

## Additional Information

**How to cite this article**: Yin, R. *et al*. miR-706 inhibits the oxidative stress-induced activation of PKCα/TAOK1 in liver fibrogenesis. *Sci. Rep.* **6**, 37509; doi: 10.1038/srep37509 (2016).

**Publisher's note:** Springer Nature remains neutral with regard to jurisdictional claims in published maps and institutional affiliations.

## Supplementary Material

Supplementary Dataset 1

Supplementary Dataset 2

## Figures and Tables

**Table 1:** Sequences of RNA and DNA Oligonucleotides.

Name	Sense Strand/Sense Primer (5′-3′)	Sense Strand/Sense Primer (5′-3′)
miRNA and siRNA duplexes		
miR-706	AGAGAAACCCUGUCUCAAAAAA	UGAGACAGGGUUUCUCUUUUU
miR-NC	UUGUACUACACAAAAGUACUG	GUACUUUUGUGUAGUACAAUU
anti-miR-706	UUUUUUGAGACAGGGUUUCUCU	
anti-NC	CAGUACUUUUGUGUAGUACAA	
siTaok1 (human)	GCAGUAUGGGAGUCCGCAATT	UUGCGGACUCCCAUACUGCTT
siPrkca (human)	AGGAACCACAAGCAGUATT	UACUGCUUGUGGUUCCUUATT
miR-706 Agomir	AGAGAAACCCUGUCUCAAAAAA	UUUUGAGACAGGGUUUCUCUUU
miR-706 Agomir-NC	UUGUACUACACAAAAGUACUG	GUACUUUUGUGUAGUACAAUU
miR-706 Antagomir	UUUUUUGAGACAGGGUUUCUCU	
miR-706 Antagomir-NC	CAGUACUUUUGUGUAGUACAA	
Primers for qPCR or RT-PCR		
HNF-4 (mouse)	CTGAAGGTGCCAACCTCAAT	CACATTGTCGGCTAAACCTG
α-SMA (mouse)	TTCCTTCGTGACTACTGCTGAG	CAATGAAAGATGGCTGGAAGAG
α-SMA (human)	ATCAAGGAGAAACTGTGTTATGTAG	GATGAAGGATGGCTGGAACAGGGTC
GAPDH (mouse)	AACTTTGGCATTGTGGAAGG	CACATTGGGGGTAGGAACAC
GAPDH (human)	GAGTCAACGGATTTGGTCGT	GACAAGCTTCCCGTTCTCAG
Bad (mouse)	AAGTCCGATCCCGGAATCC	GCTCACTCGGCTCAAACTCT
Bad (human)	GAGCCCGGGGTGCTGGAGGGA	GGCGGCACAGACGCGGGCTTT
Bak1 (mouse)	GTCAGGCAGGTGACAAGTGAG	TTAGTCCAGGCAGTCATGTGG
Bak1 (human)	GCTCCCAACCCATTCACTAC	TCCCTACTCCTTTTCCCTGA
Bax (human)	ATGGACGGGTCCGGGGAGCA	CCCAGTTGAAGTTGCCGTCA
Bax (mouse)	TGAAGACAGGGGCCTTTTTG	AATTCGCCGGAGACACTCG
Desmin (mouse)	AGGAGAGCAGGATCAACCTT	CCTGGCTTACAGCACTTCAT
Bbc3 (human)	GGACGACCTCAACGCACAGT	AATTGGGCTCCATCTCGGGG
Bbc3 (mouse)	AGCAGCACTTAGAGTCGCC	CCTGGGTAAGGGGAGGAGT
Col1a1(mouse)	GCTCCTCTTAGGGGCCACT	CCACGTCTCACCATTGGGG
Col1a1(human)	AGCCAGCAGATCGAGAACAT	CAGTTCTTCTGGGCCACACT
U6	CTCGCTTCGGCAGCACA	AACGCTTCACGAATTTGCGT
18S	AAACGGCTACCACATCCAAG	CCTCCAATGGATCCTCGTTA
mmu-miR-706-LP	CTCAACTGGTGTCGTGGAGTCGGCAATTCAGTTGAGTTTTTTGA	
mmu-miR-706-PF	ACACTCCAGCTGGGACAGAAACCCTGTCTC	
mmu-miR-706-PR	TGGTGTCGTGGAGTCG	
Primers for cloning (Restriction enzyme sites were underlined)		
miR-706	AGAGAAACCCUGUCUCAAAAAA	
Prkca-3′UTR	CCACCUGACAGUCAUUUUCUCAC	
Taok1-3′UTR	GUUAUAUGGAAACAGGUUUCUCU	
