# Supplementary material for: miR-706 inhibits the oxidative stress-induced activation of PKCα/TAOK1 in liver fibrogenesis
Source: Sci Rep. 2016 Nov 23;6:37509. doi: 10.1038/srep37509 (PMC5120320; doi:10.1038/srep37509)
Supplement: Supplementary Dataset 1 [file srep37509-s1.doc]

**Title page**

miR-706 inhibits the oxidative stress-induced activation of PKCα/TAOK1 in liver fibrogenesis

Ruili Yin^1^, Duo Guo^1^, Shuxian Zhang^1^, Xiuying Zhang^1^*

^1^Department of Histology and Embryology, School of Basic Medical Sciences, Capital Medical University

Ruili Yin and Duo Guo contributed equally to this work

Corresponding Author:

Dr. Xiuying Zhang

Department of Histology and Embryology

School of Basic Medical Sciences, Capital Medical University

10 Xi tou tiao, You An Men Wai, 100069, Beijing, China

T: (86)18612536738. E-mail: zhxy0515@hotmail.com

**Supporting Figure 1**

**
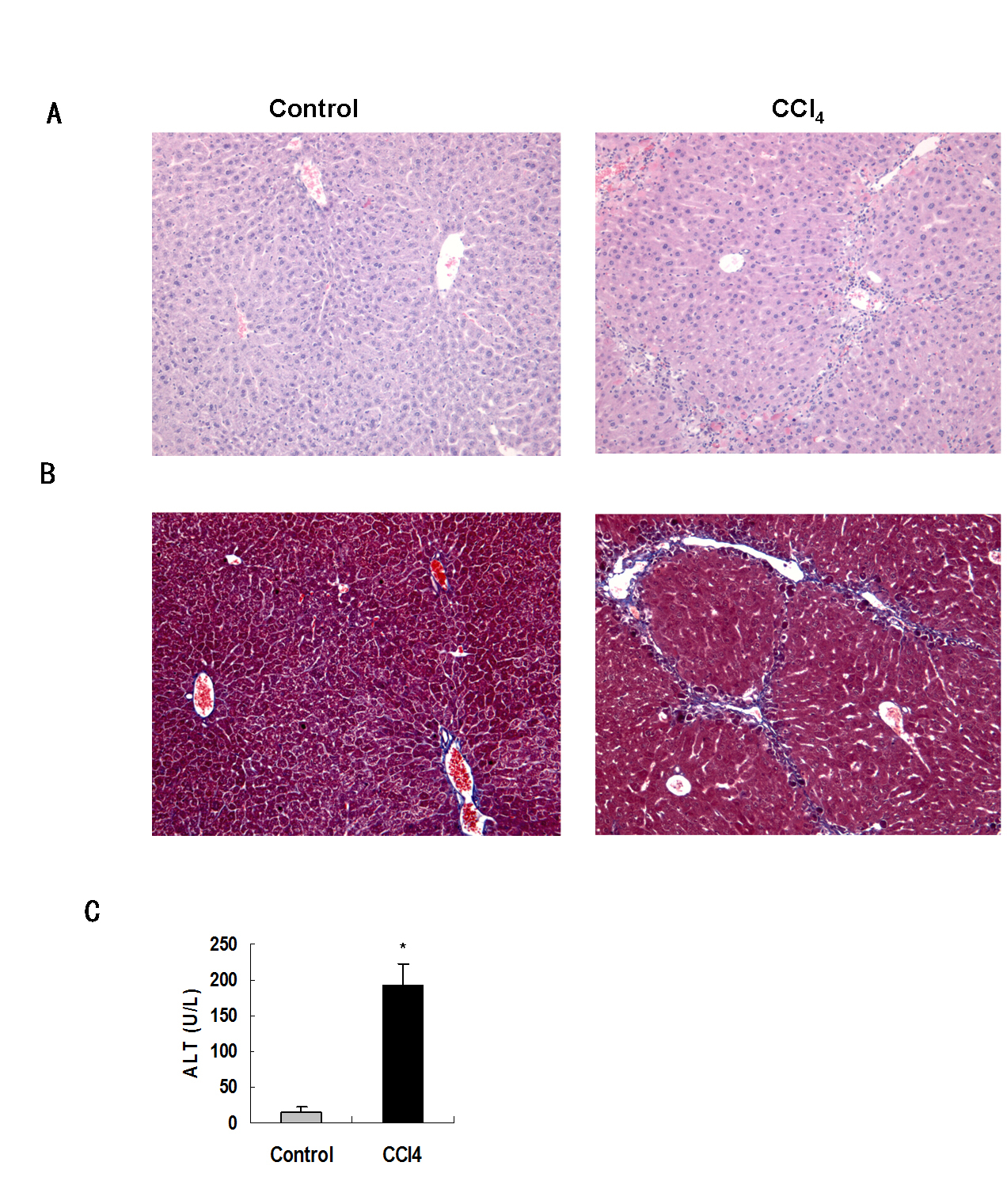
**

**Supporting Figure 2**

**
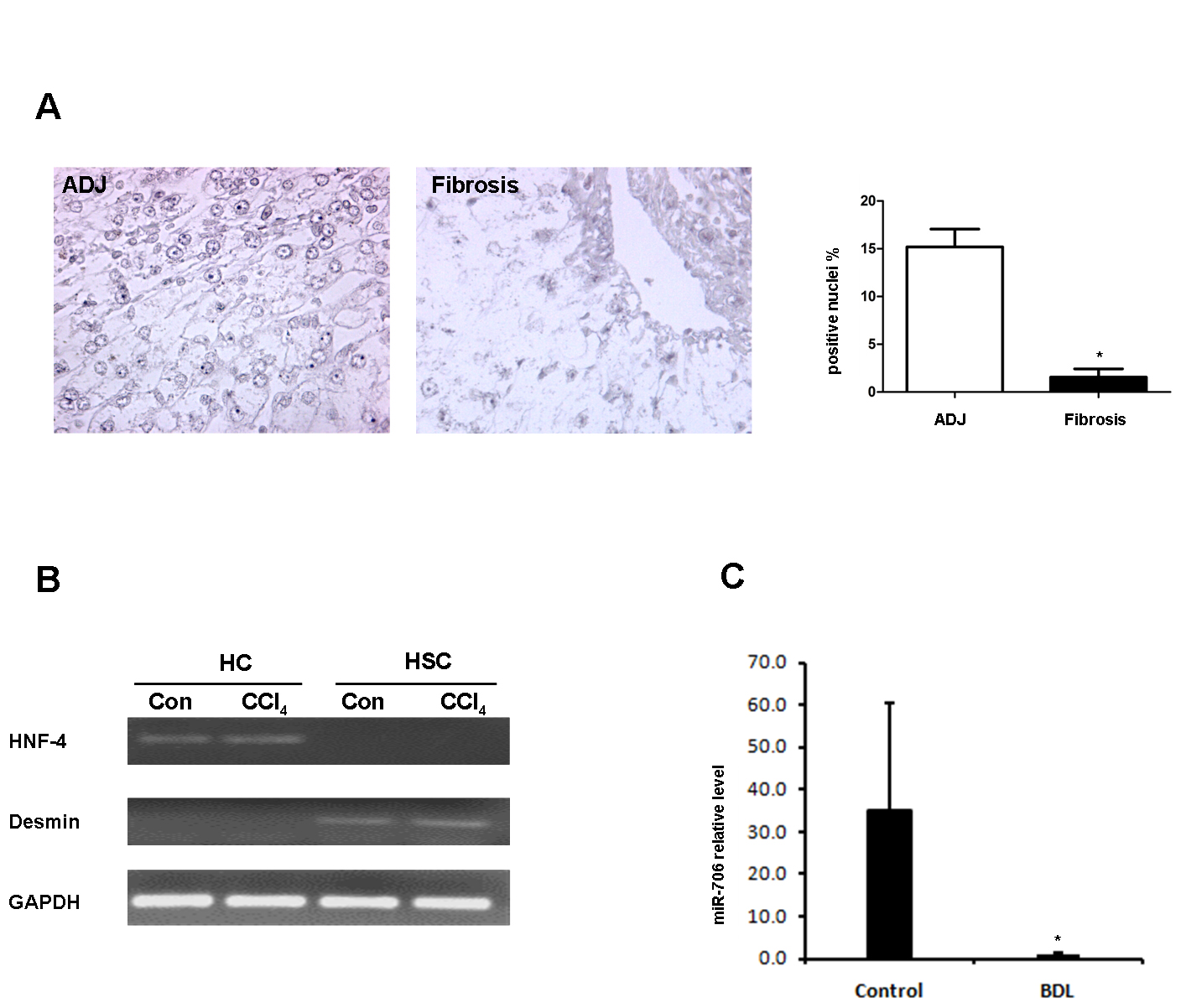
**

**Supporting Figure 3**

**
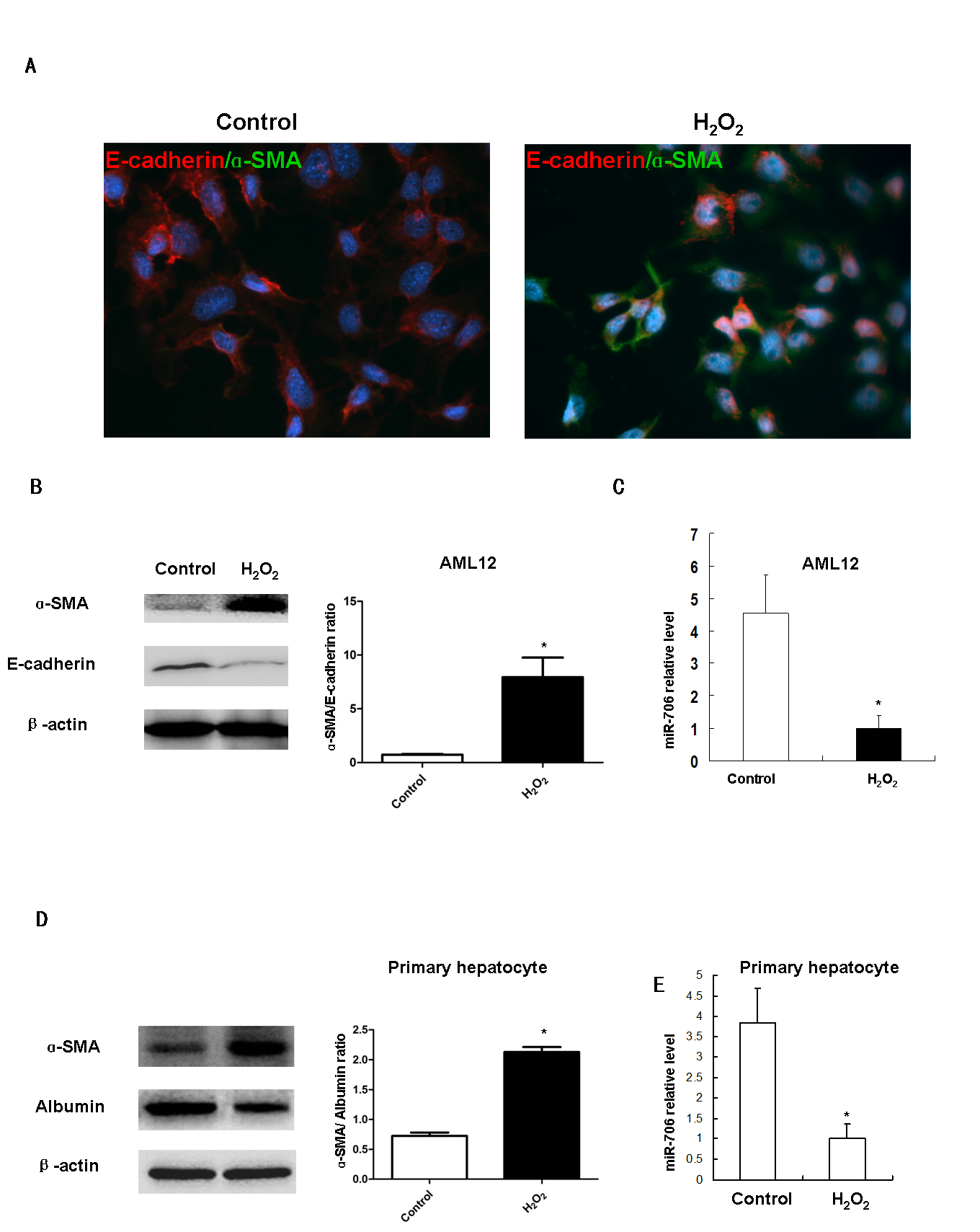
**

**Supporting Figure 4**

**
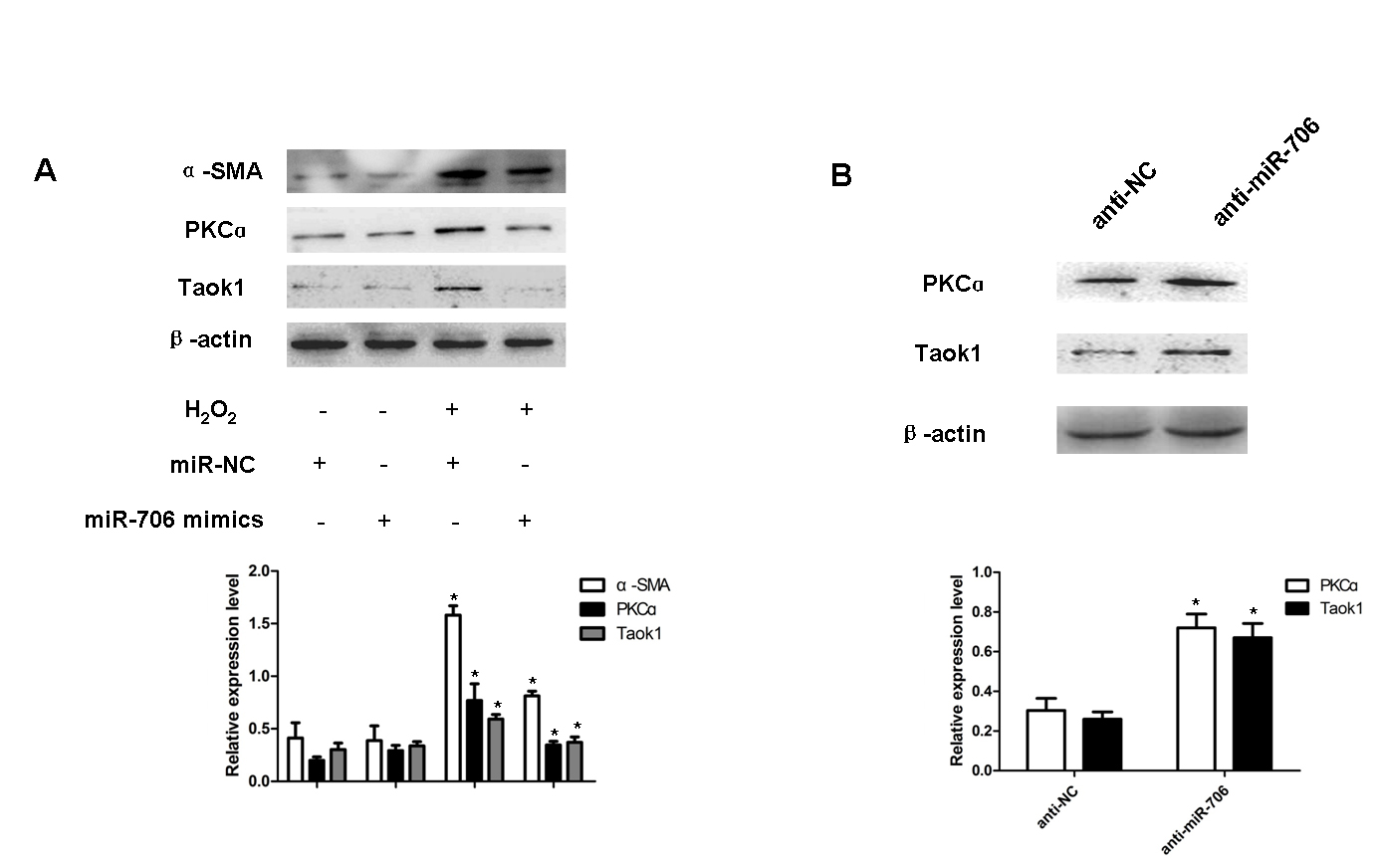
**

**Supporting Figure 5**

**
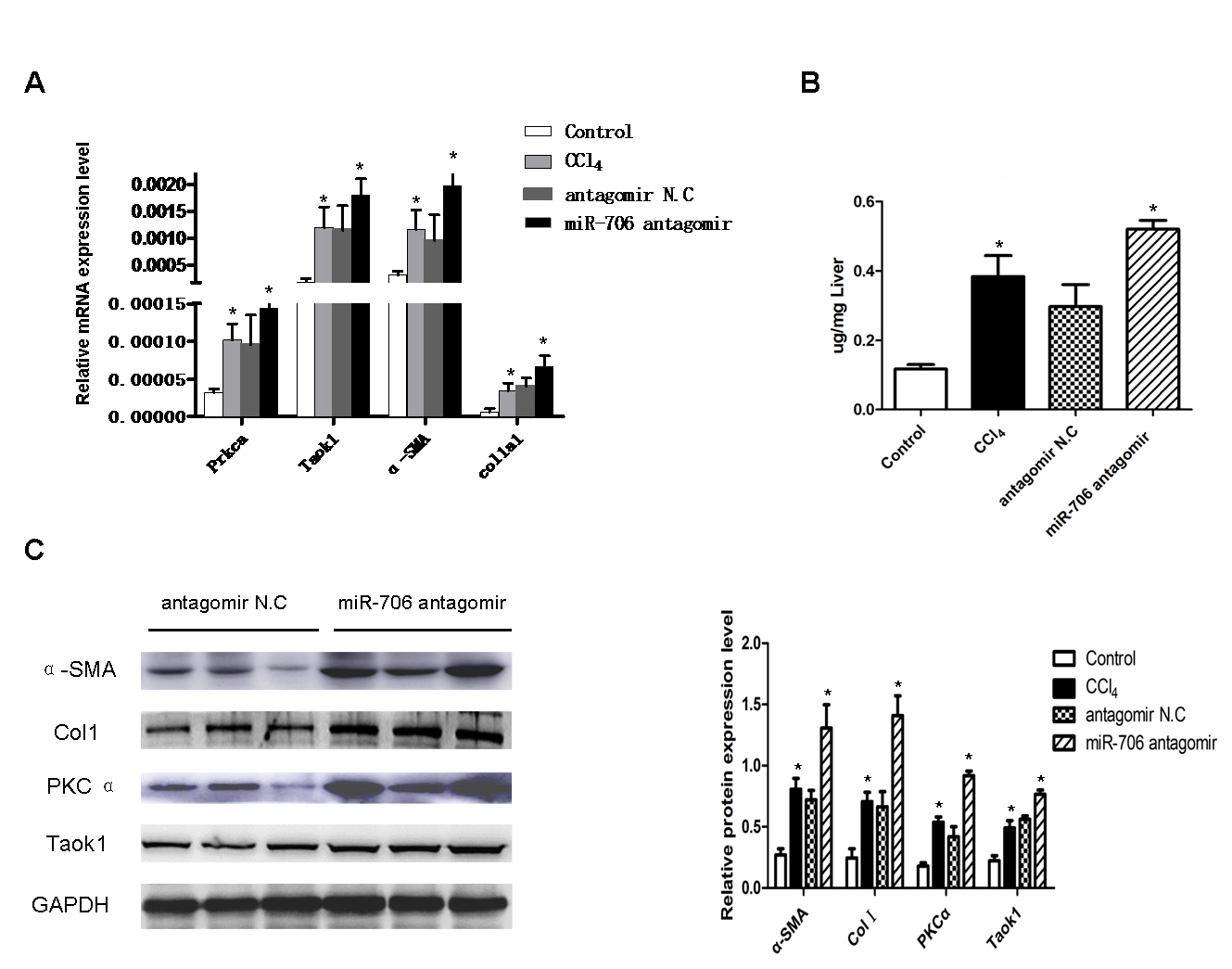
**

**SUPPORTING MATERIALS AND METHODS**

**Bile duct ligation**

BDL and sham (control) surgery (n=10 in each group) were performed on C57BL/6J mice (8 weeks old) under pentobarbital sodium (50 mg/kg) anesthesia. In the BDL operation, a laparotomy was performed after midline laparotomy. The common bile duct was ligated three times with 5–0 silk and transected between the two most distal ligations. Sham operation was performed similarly, except for ligation and transection of the bile duct (0 h, n = 5). Sham-operated animals without BDL served as controls (n = 5). All surgical procedures were performed under aseptic conditions. Mice were sacrificed at 4 weeks (wk) after surgery. The livers were rapidly removed and briefly washed in PBS, and then total RNAs were isolated from the liver samples using RNAlater solution, and the expression of miR-706 in the liver was measured by real-time PCR.

**Supporting Figure legends**

**Supporting Figure 1. CCl_4_ treatment induces liver fibrosis.** Representative photographs of H&E (**A**) and Masson’s staining (**B**) of liver sections from 6-week CCl_4_-treated and control mice. (**C**) Blood serum levels of ALT (U/L) were detected in 6-week CCl_4_-treated and control mice; * *P* *<*0.05, n=4 in each group.

**Supporting Figure 2.** **(A)** Representative photograph of miR-706 localization in human liver adjacent to hepatocellular carcinoma (left panel) and liver fibrosis (right panel) detected by *in situ* hybridization. ADJ: human liver adjacent to hepatocellular carcinoma, Fibrosis: fibrotic liver. * *P* *<*0.05, n=8 in Adjacent liver, n=12 in Fibrosis. (**B**) Cell-specific markers in isolated mice hepatocytes and HSCs. Hepatocytes and HSCs were isolated and analyzed for HNF-4 (a specific marker of hepatocytes) and desmin (a typical marker for HSCs) by RT-PCR analysis. GAPDH was used as an internal control. Hc : hepatocytes. (C) miR-706 expression in bile duct ligation liver. * *P* *<*0.05, n=5 in each group.

**Supporting Figure 3**. The murine hepatocyte cell line AML12 and primary hepatocyte were treated with 300 μM H_2_O_2_ for 48 h. Protein levels of E-cadherin and α-SMA were determined by dual-immunofluorescence staining (**A**), and (**B**) Western blot, (**D**) Protein levels of Albumin and α-SMA were determined by Western blot, β–actin was used as loading control. (**C, E**) miR-706 expression was examined in AML12 (**C)** and primary hepatocyte (**E)** by RT-PCR, * *P* *<*0.05.

**Supporting Figure 4.** (**A**) **miR-706 inhibits oxidative stress-induced fibrotic related genes in AML12 cells**. Introduction of miR-706 repressed H_2_O_2_-stimulated expression of α-SMA, PKCα, and TAOK1. AML12 cells were transfected with negative control (NC) or miR-706 duplex for 8 h, and then stimulated with 300 μM H_2_O_2_ or remained untreated for 48 h before immunoblotting analysis for α-SMA, PKCα and TAOK1. β–actin was used as an internal control for immunoblotting. * *P* *<*0.05. (**B**) Knockdown of endogenous miR-706 enhanced PKCα and TAOK1 protein levels. L02 cells were transfected with the inhibitor of miR-706 (anti-miR-706) or its negative control (anti-NC) for 48 h before immunoblotting. The PKCα and TAOK1 levels were normalized to that of β–actin (internal control). * *P* *<*0.05.

**Supporting Figure 5. Inhibition of miR-706 aggravated CCl_4_-induced liver fibrosis.** C57/BL6 mice were administered with CCl_4_ twice weekly by intraperitoneal injection for 6 weeks to induce liver fibrosis. Separately, CCl_4_-treated mice were injected with miR-706 antagomir or miR-706 antagomir negative control (antagomir N.C) by tail vein injection once per week after 4 weeks of CCl_4_ treatment. (**A**) Increased prkca, TaoK1, α-SMA and Col1a1 expression were detected by real-time PCR in 6-week CCl_4_-treated livers injected with miR-706 antagomir. The levels of target genes in each sample were normalized to that of GAPDH (internal control). * *P* *<*0.05. (**B**) Quantification of hepatic hydroxyproline content; the data are expressed as hydroxyproline (μg)/liver wet weight (mg). * *P* *<*0.05. (**C**) Injection with miR-706 antagomir exacerbated the protein expression of α-SMA, Col1, PKCα and TAOK1 in liver tissue. The levels of target genes in each sample were normalized to that of GADPH (internal control). * *P* *<*0.05.
