# Supplementary material for: miR-706 inhibits the oxidative stress-induced activation of PKCα/TAOK1 in liver fibrogenesis
Source: Sci Rep. 2016 Nov 23;6:37509. doi: 10.1038/srep37509 (PMC5120320; doi:10.1038/srep37509)
Supplement: Supplementary Dataset 2 [file srep37509-s2.doc]

**Title page**

miR-706 inhibits the oxidative stress-induced activation of PKCα/TAOK1 in liver fibrogenesis

Ruili Yin^1^, Duo Guo^1^, Shuxian Zhang^1^, Xiuying Zhang^1^*

^1^Department of Histology and Embryology, School of Basic Medical Sciences, Capital Medical University

Ruili Yin and Duo Guo contributed equally to this work

Corresponding Author:

Dr. Xiuying Zhang

Department of Histology and Embryology

School of Basic Medical Sciences, Capital Medical University

10 Xi tou tiao, You An Men Wai, 100069, Beijing, China

T: (86)18612536738. E-mail: zhxy0515@hotmail.com

**Fig.3A**

3’-Nitrotyrosine


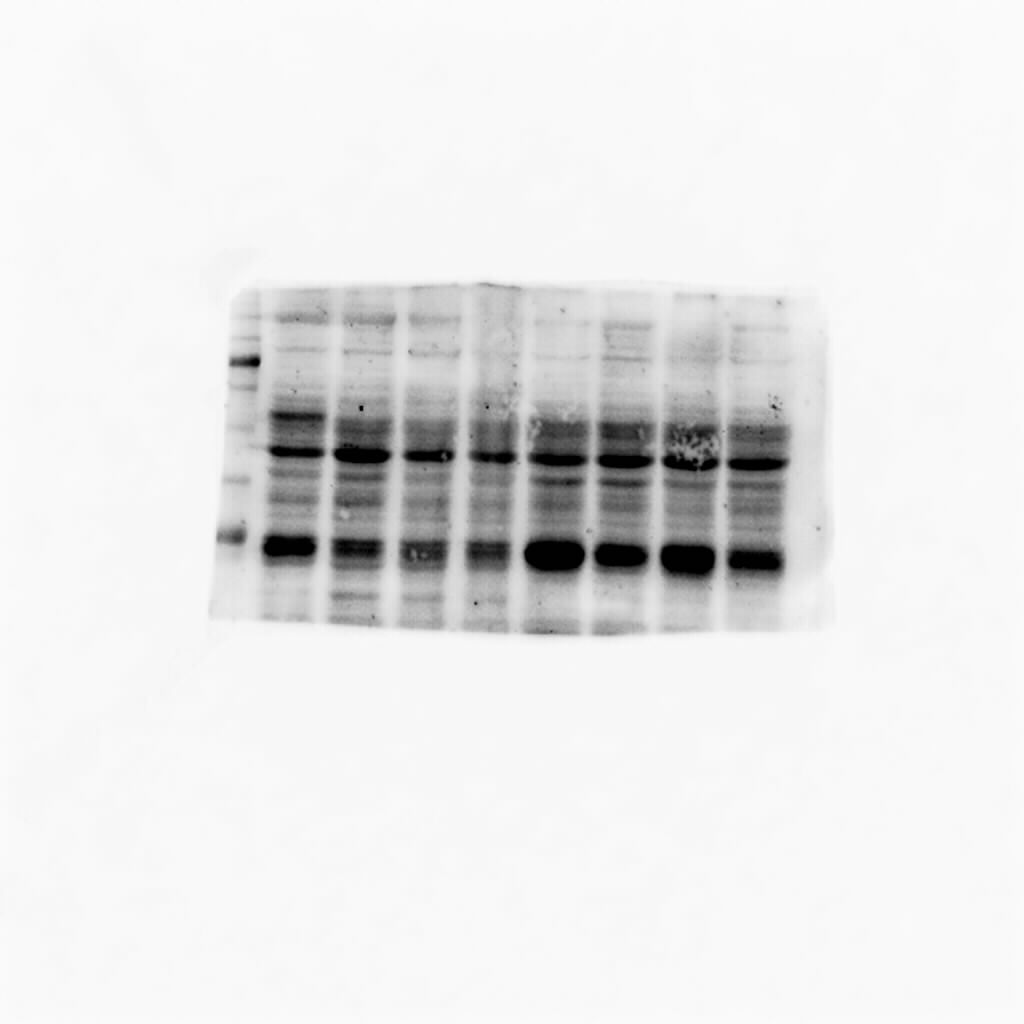


18KD

GAPDH


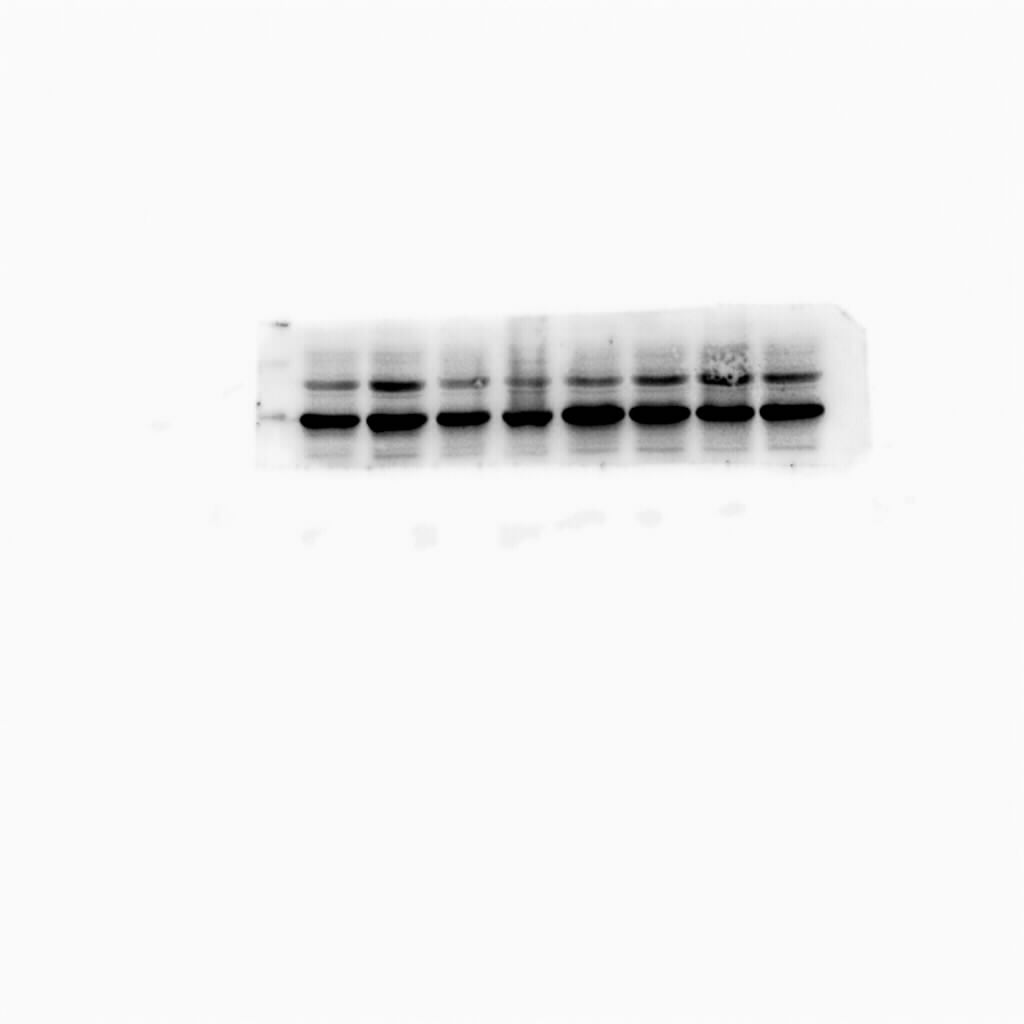


37KD

**Fig.3C**

α-SMA


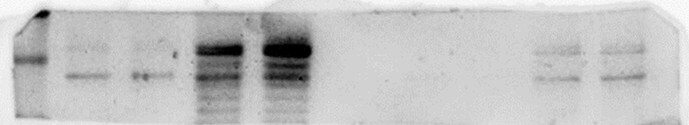


42KD

β-actin


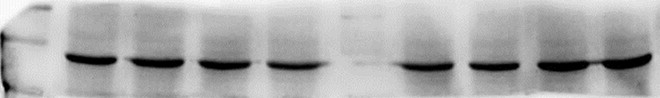


45KD

**Fig.3F**


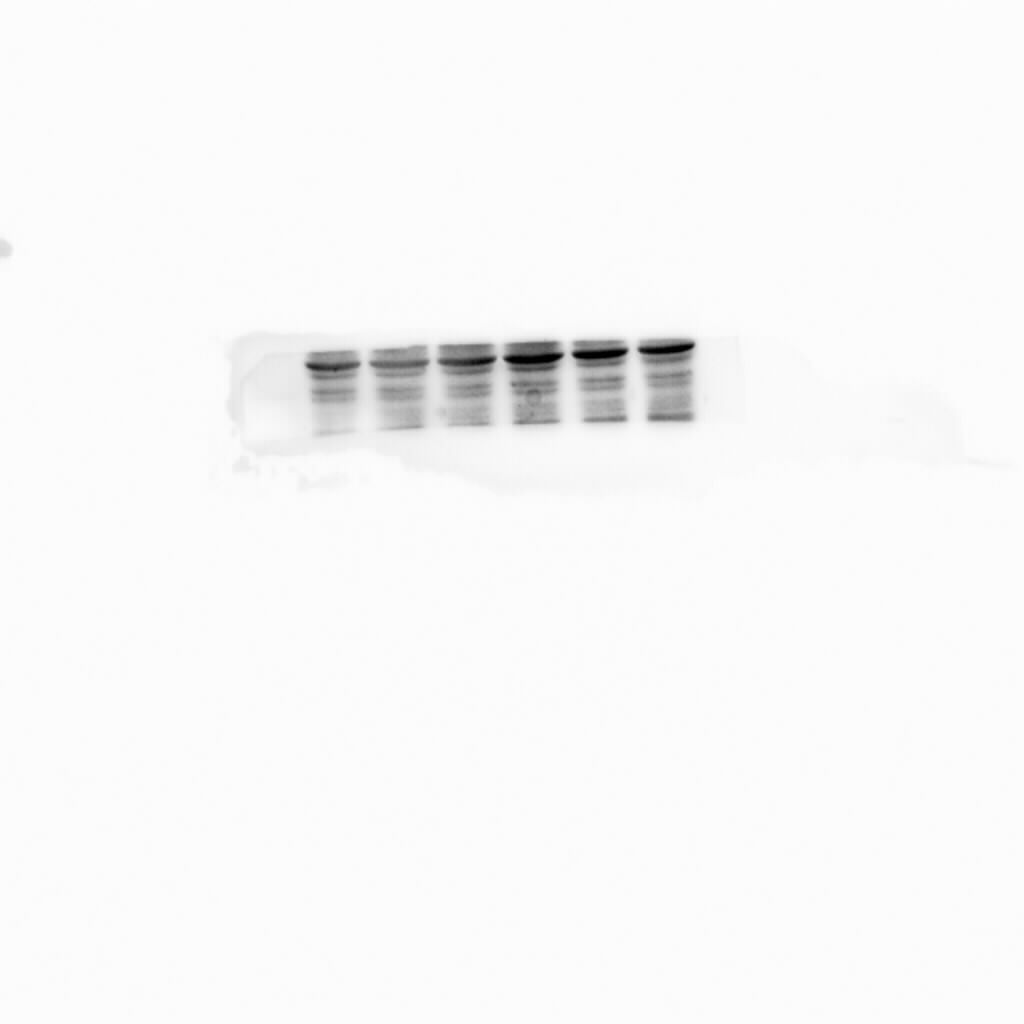
α-SMA

42KD

Albumin


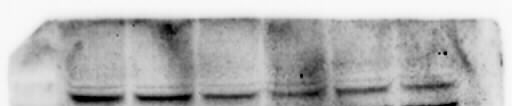


69KD

β-actin


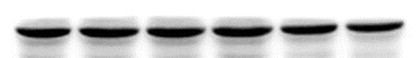


45KD

**Fig.4A**

α-SMA


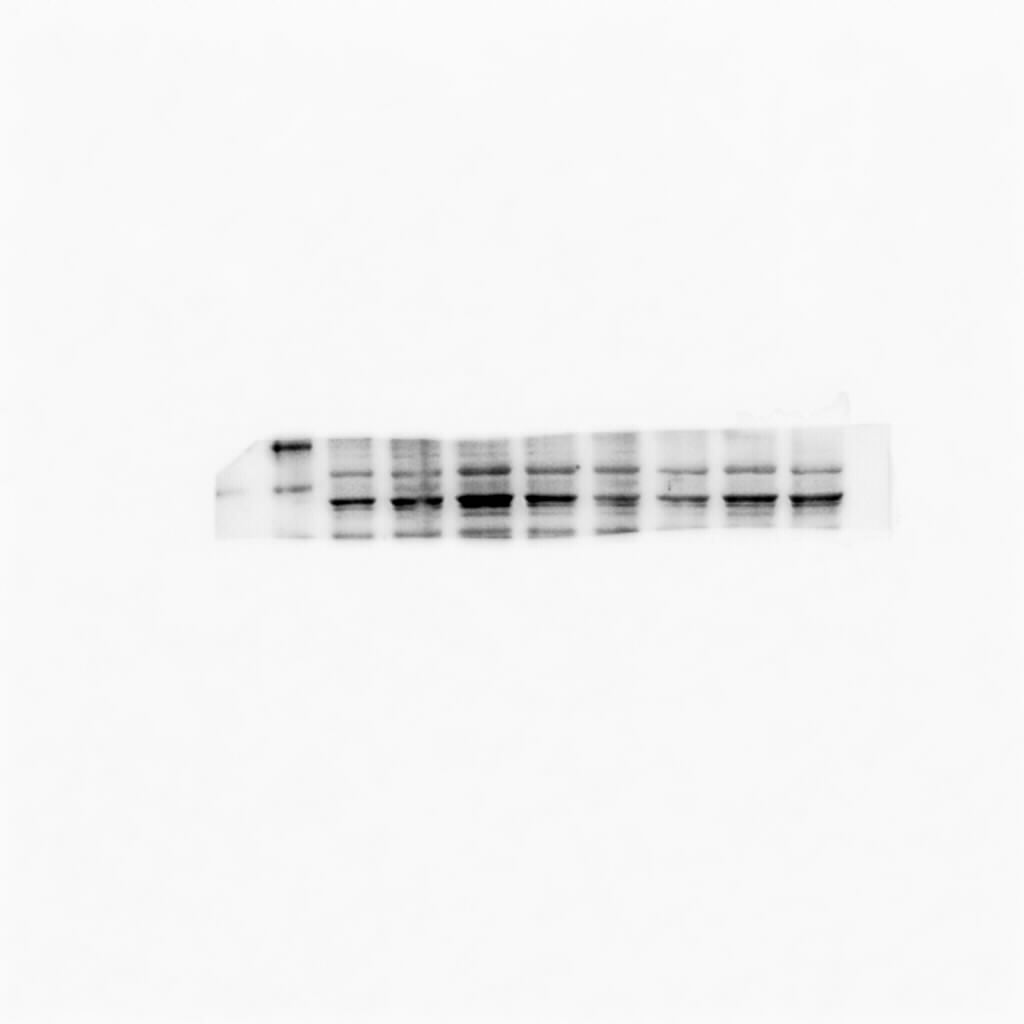


42KD

PKCα


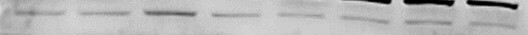


80KD

Taok1


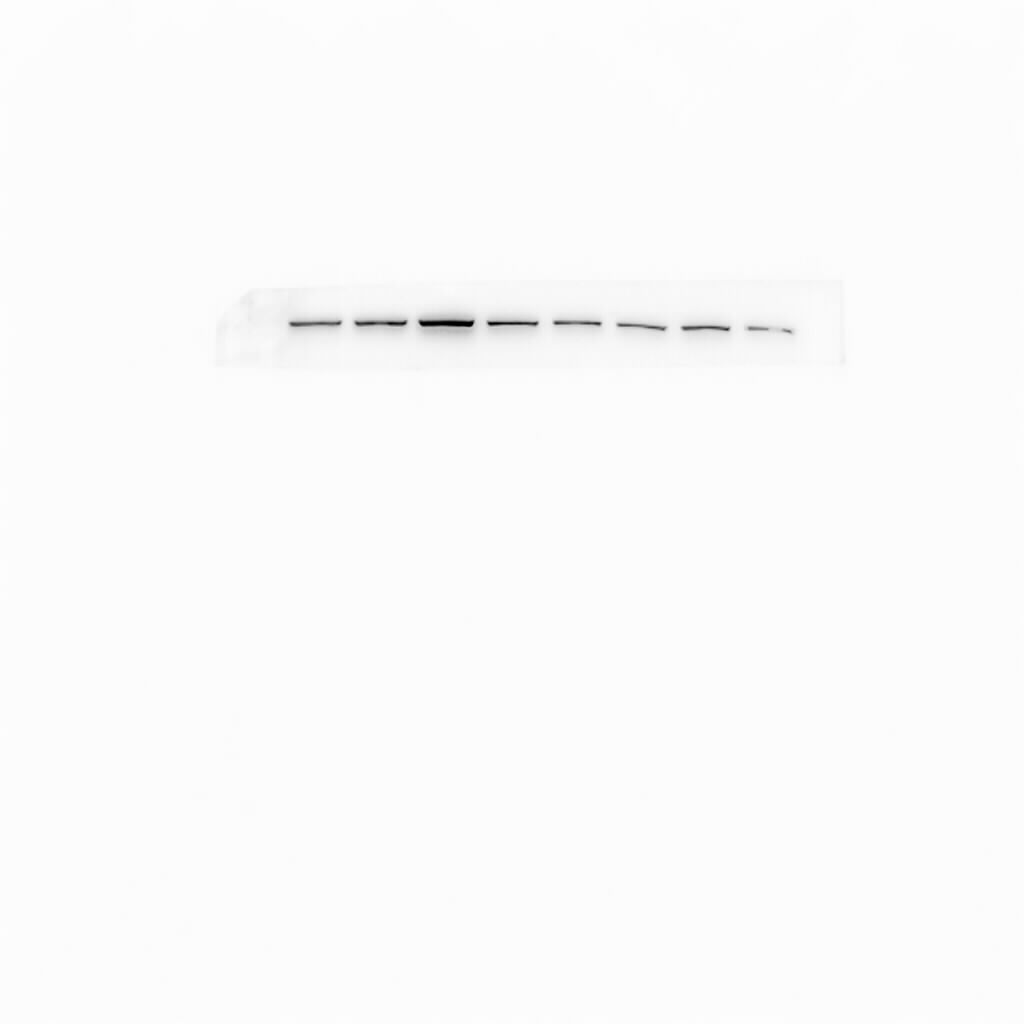


116KD

β-actin


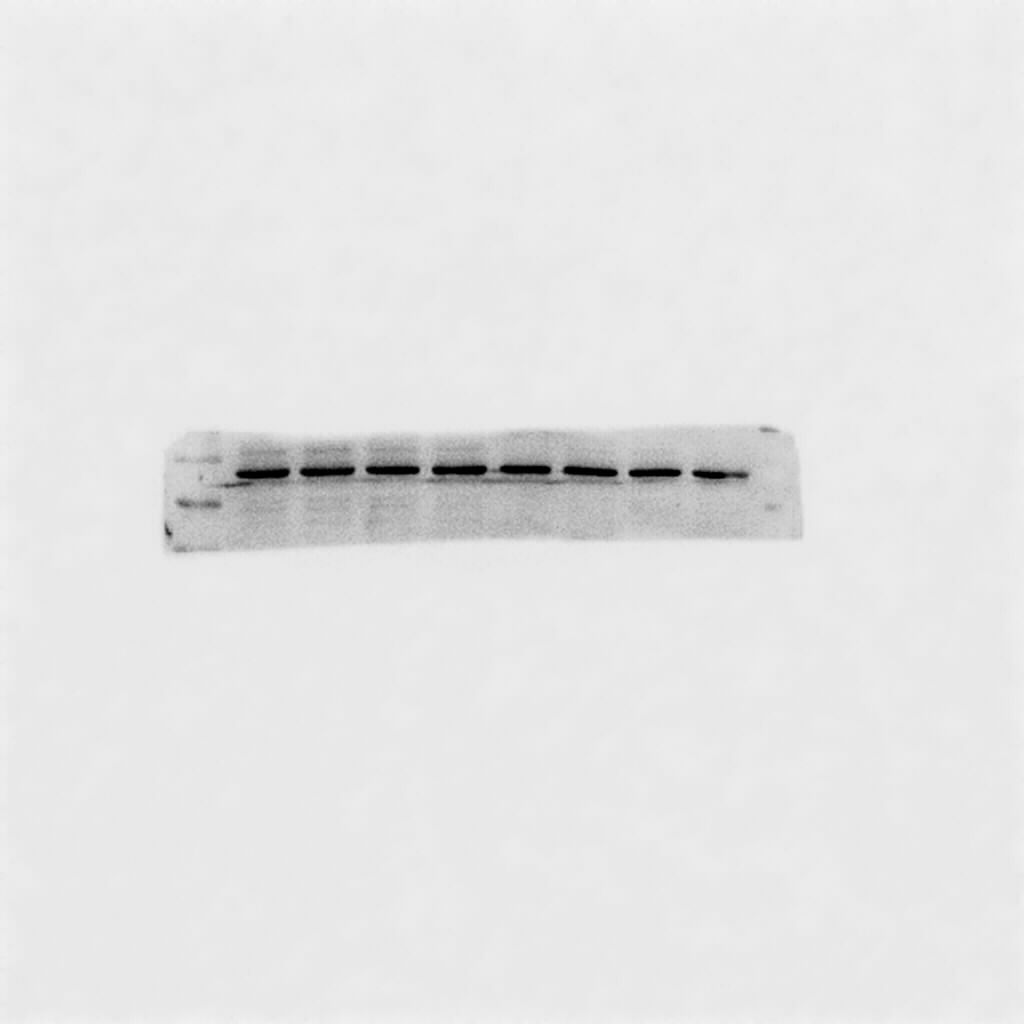


45KD

**Fig.4B**

PKCα


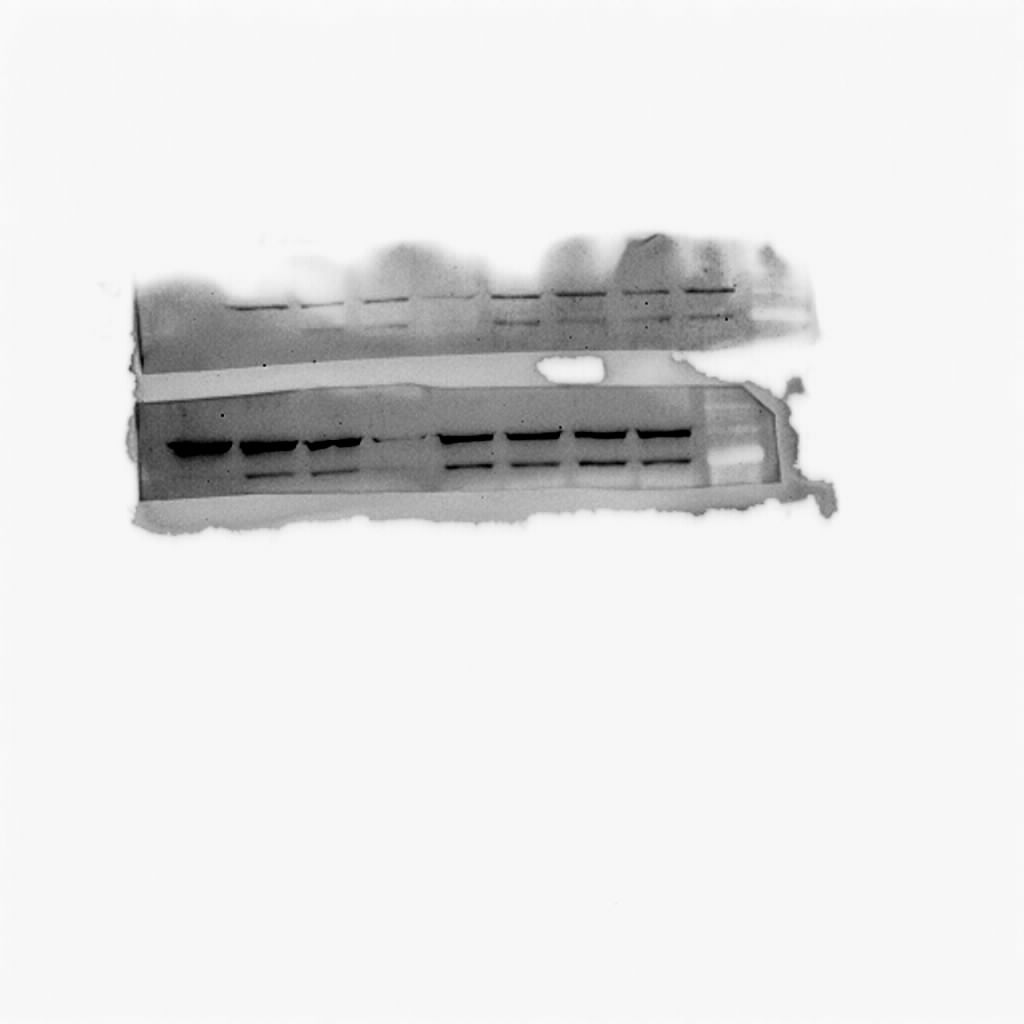


80KD


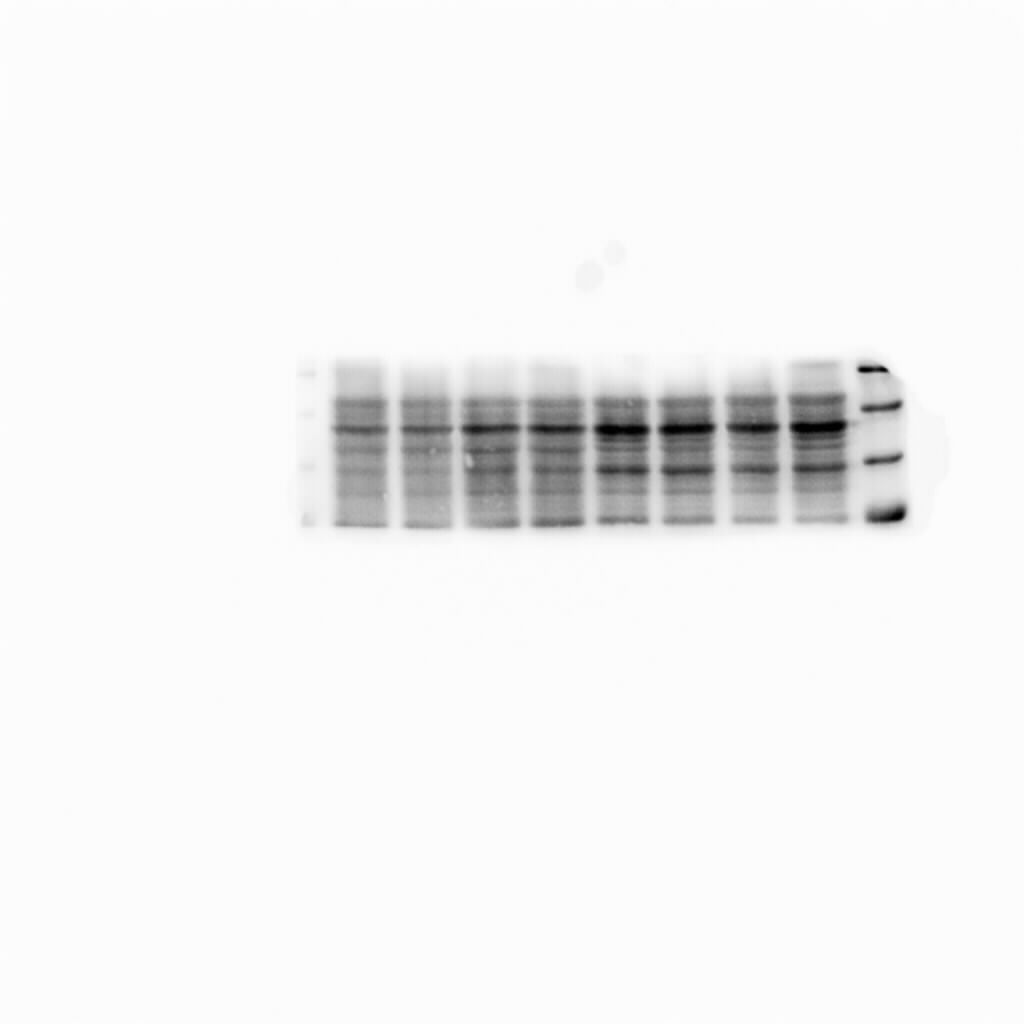
P-ERK

44KD

42KD


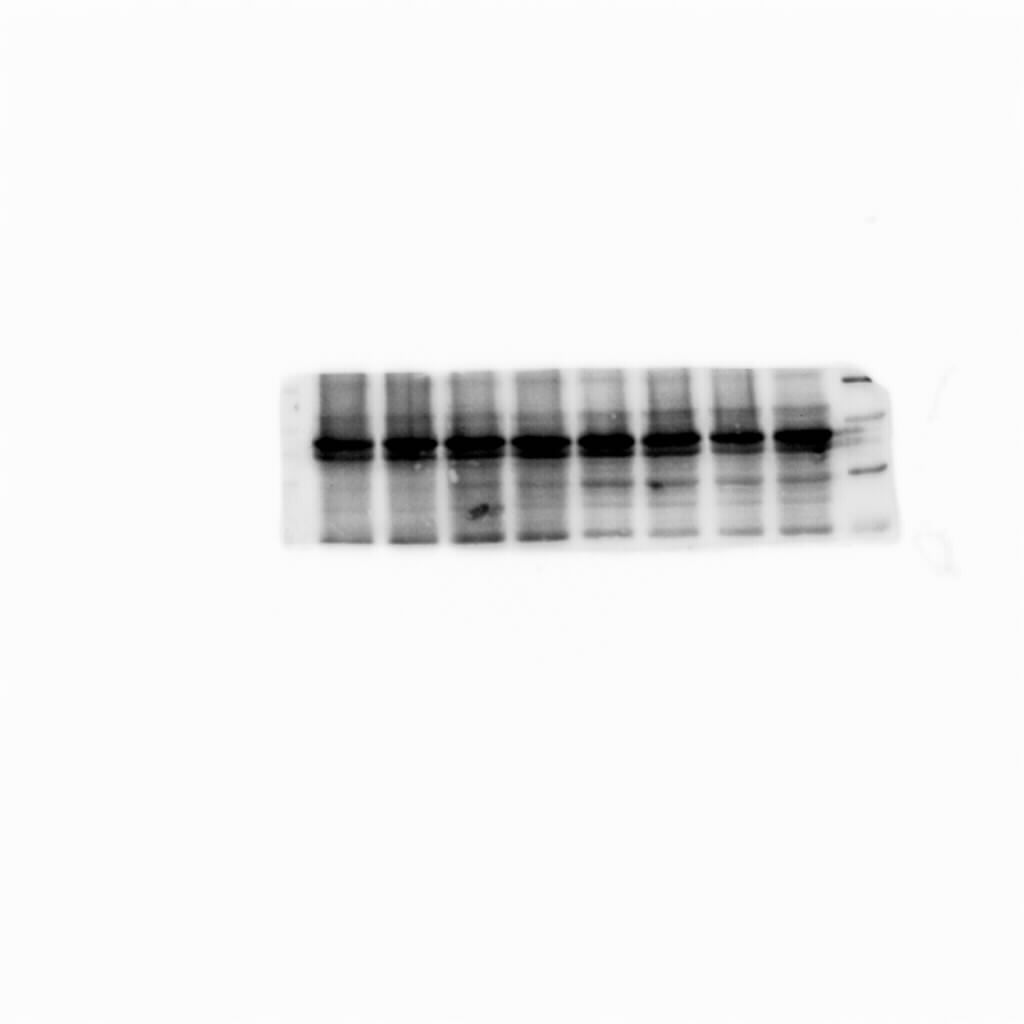
ERK

44KD

42KD


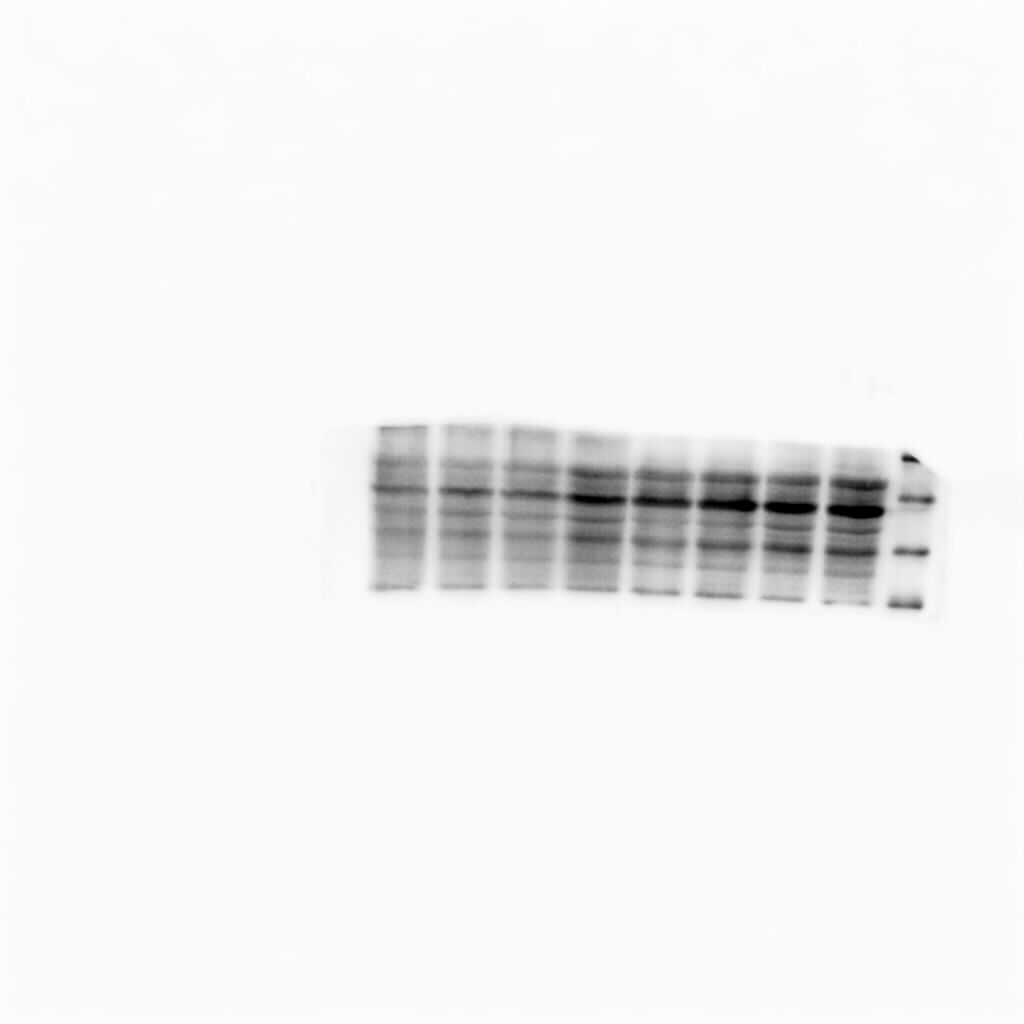
P-JNK

54KD

46KD


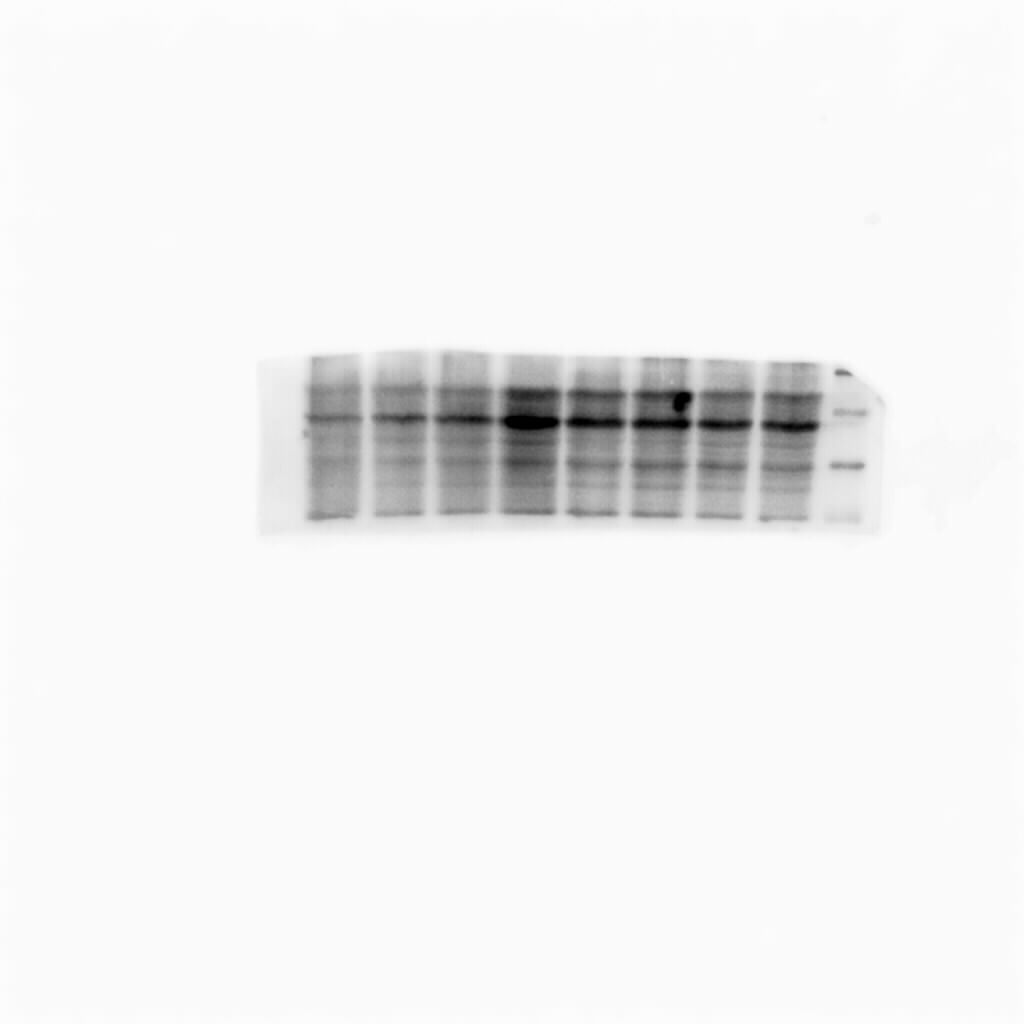
JNK

54KD

46KD

GAPDH


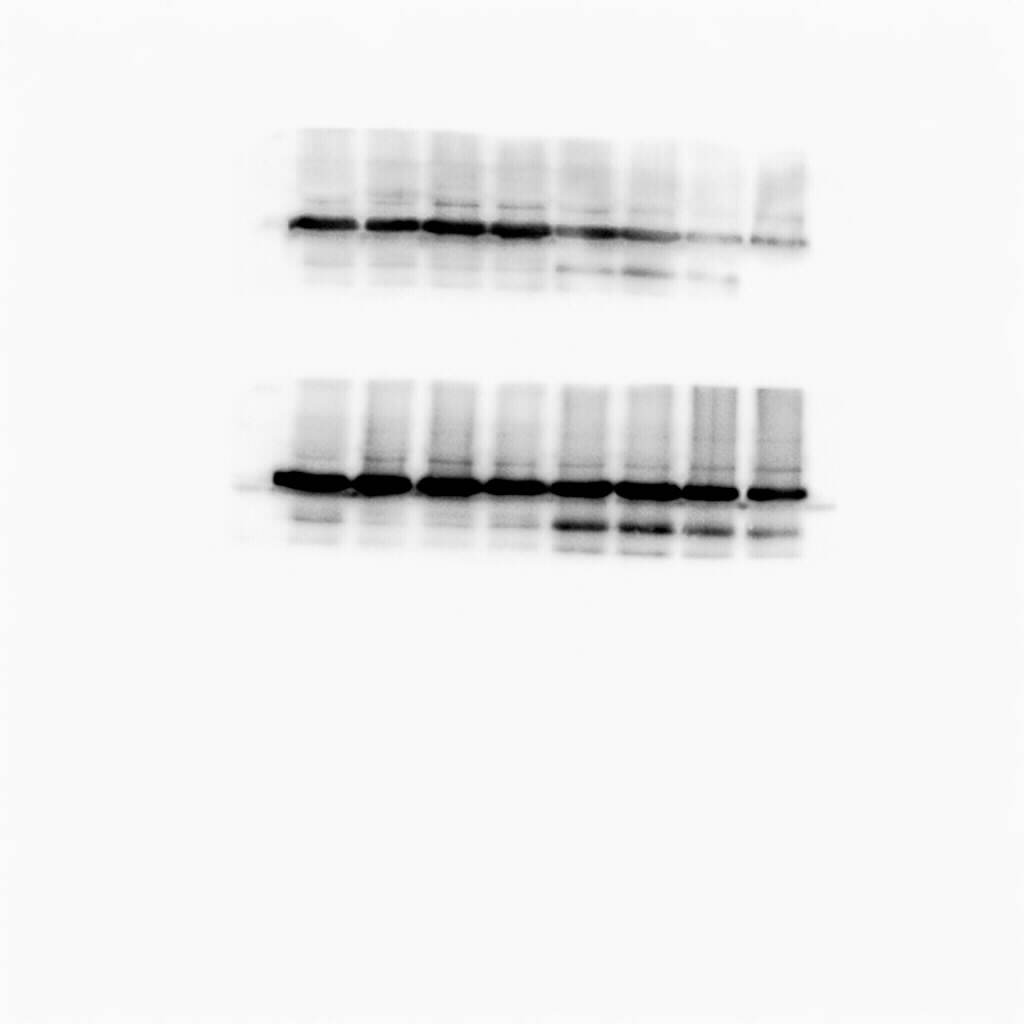


37KD

Taok1


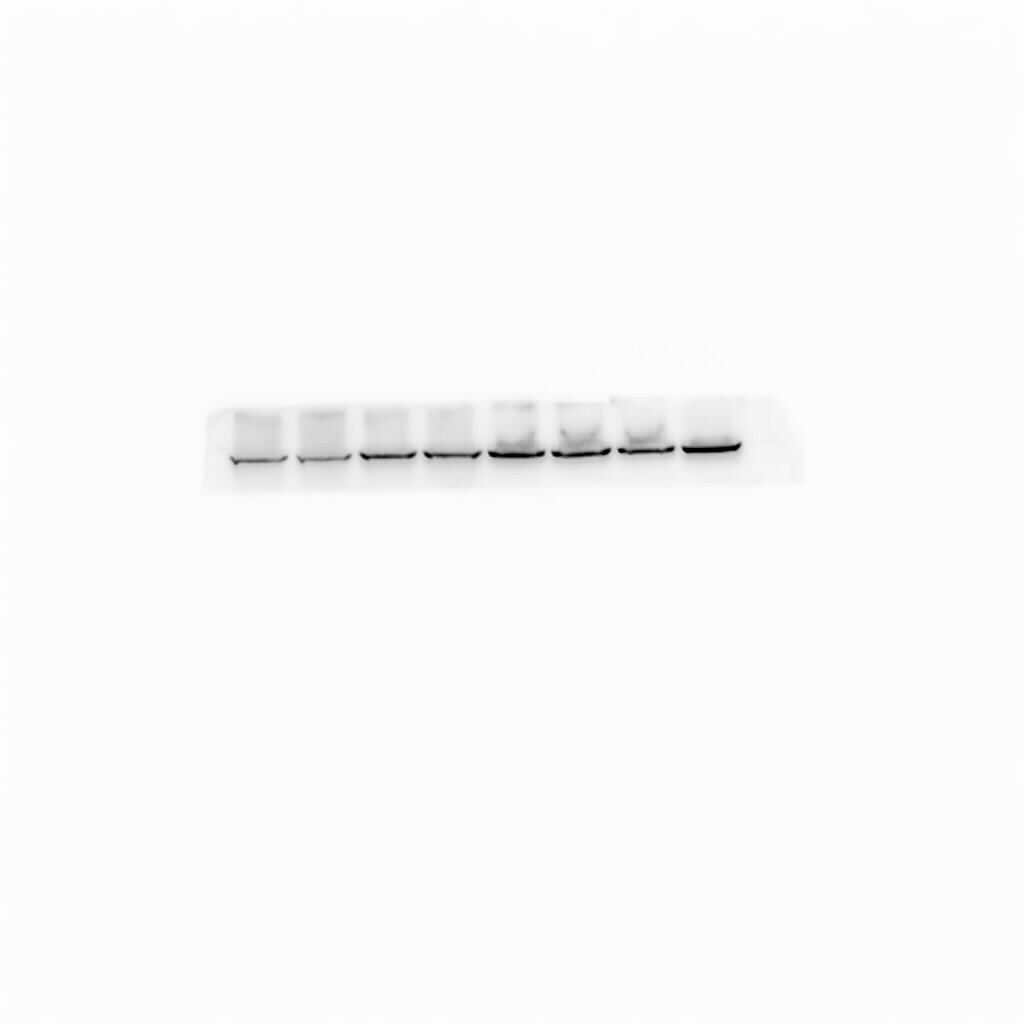


116KD

p-P38


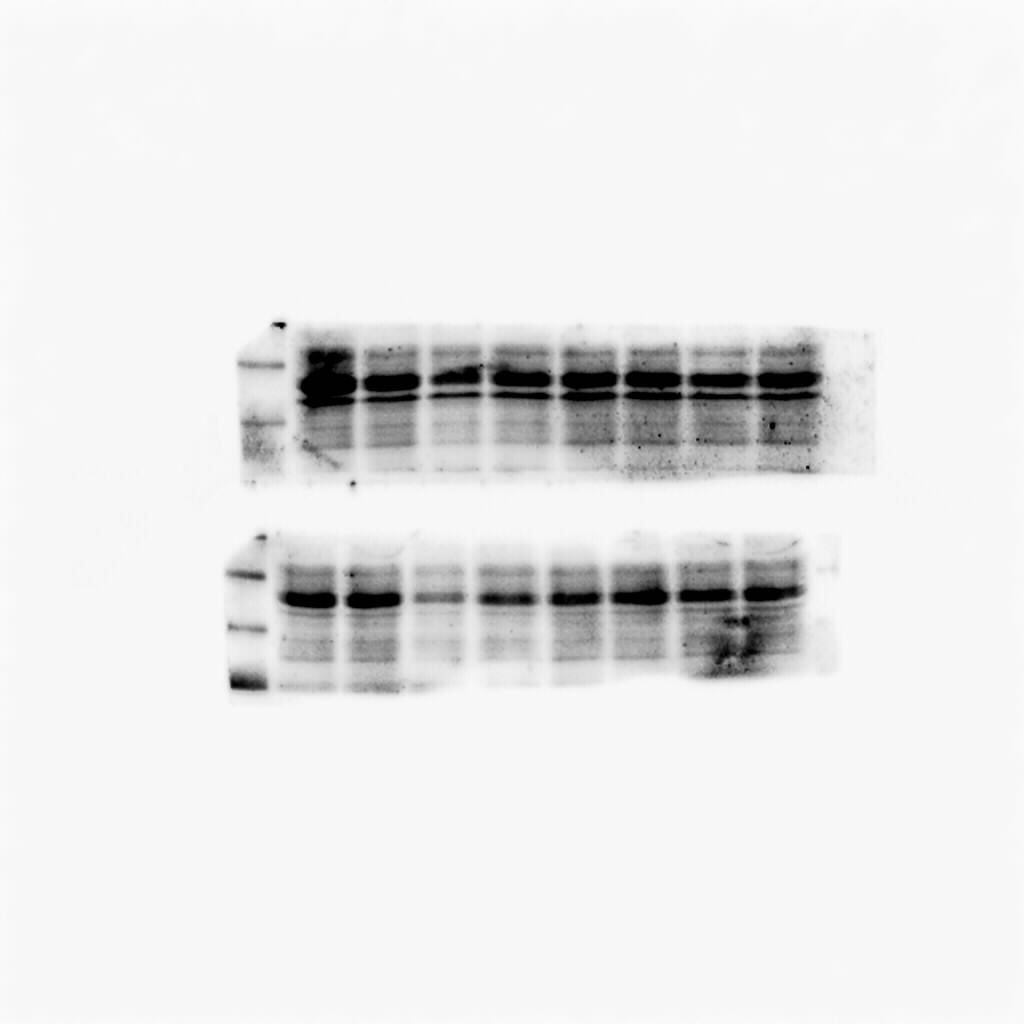


38KD

P38


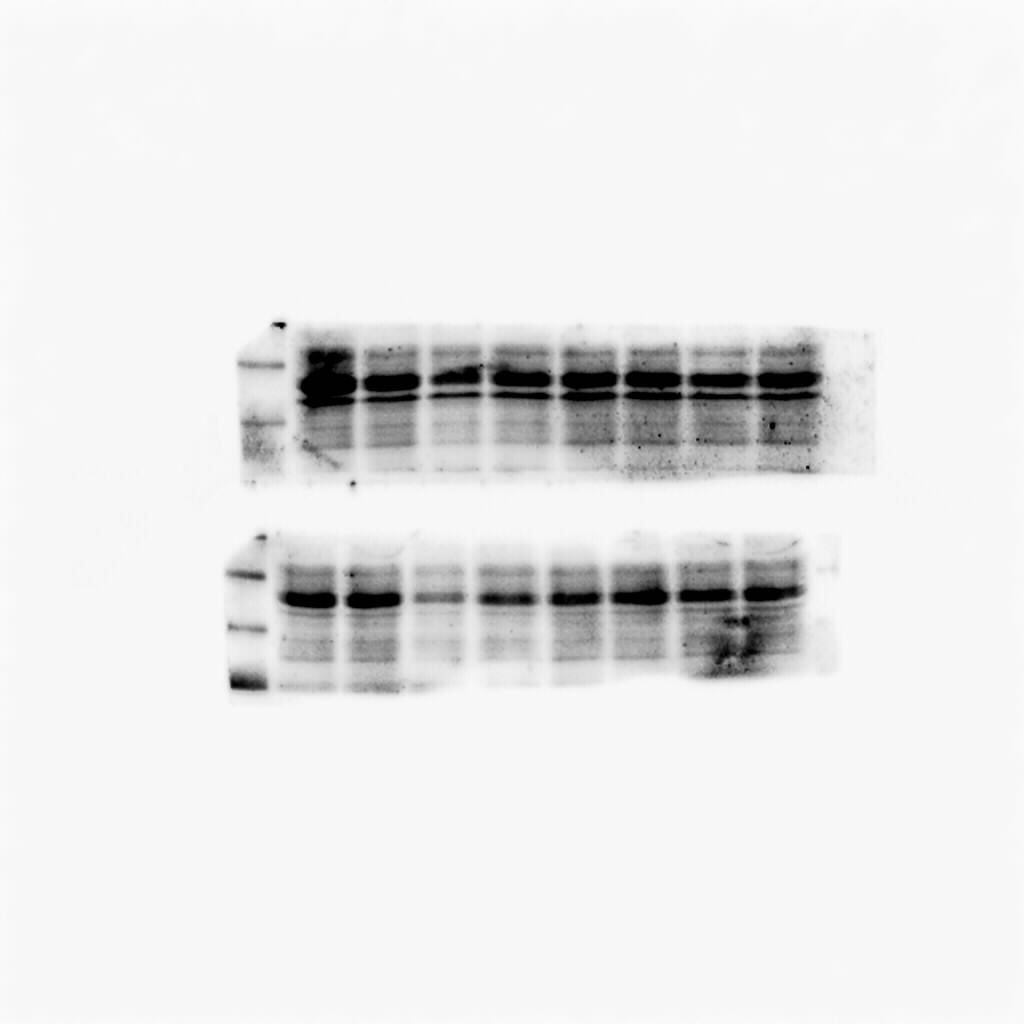


38KD

P-MEK3


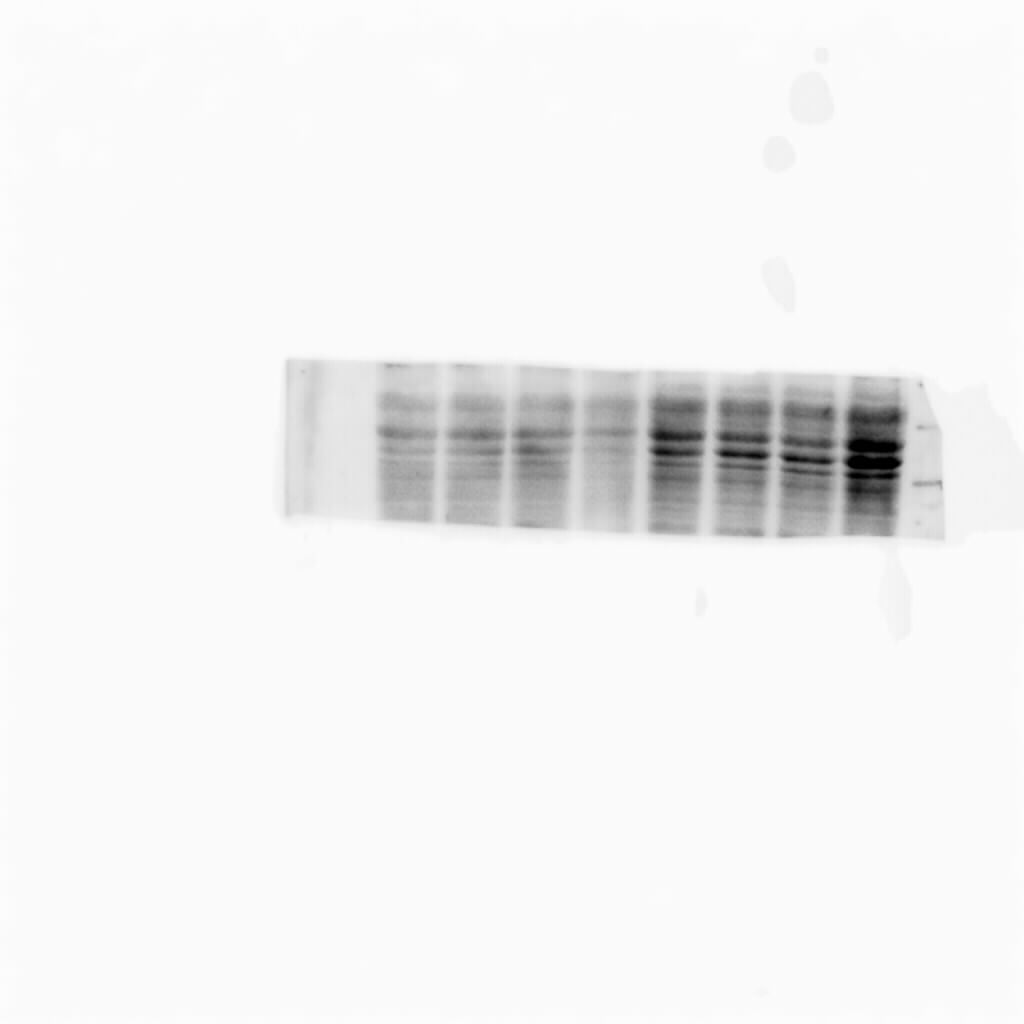


39KD

MEK3


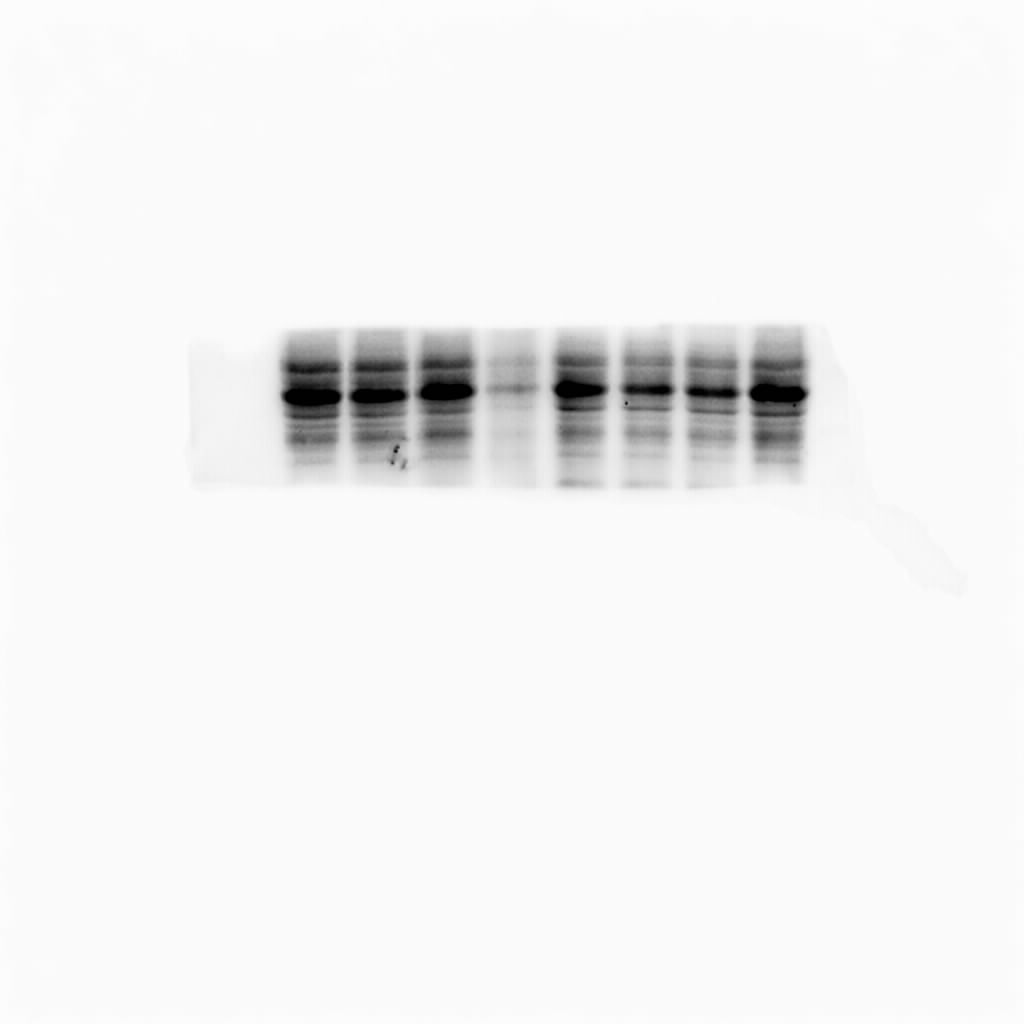


39KD

GAPDH


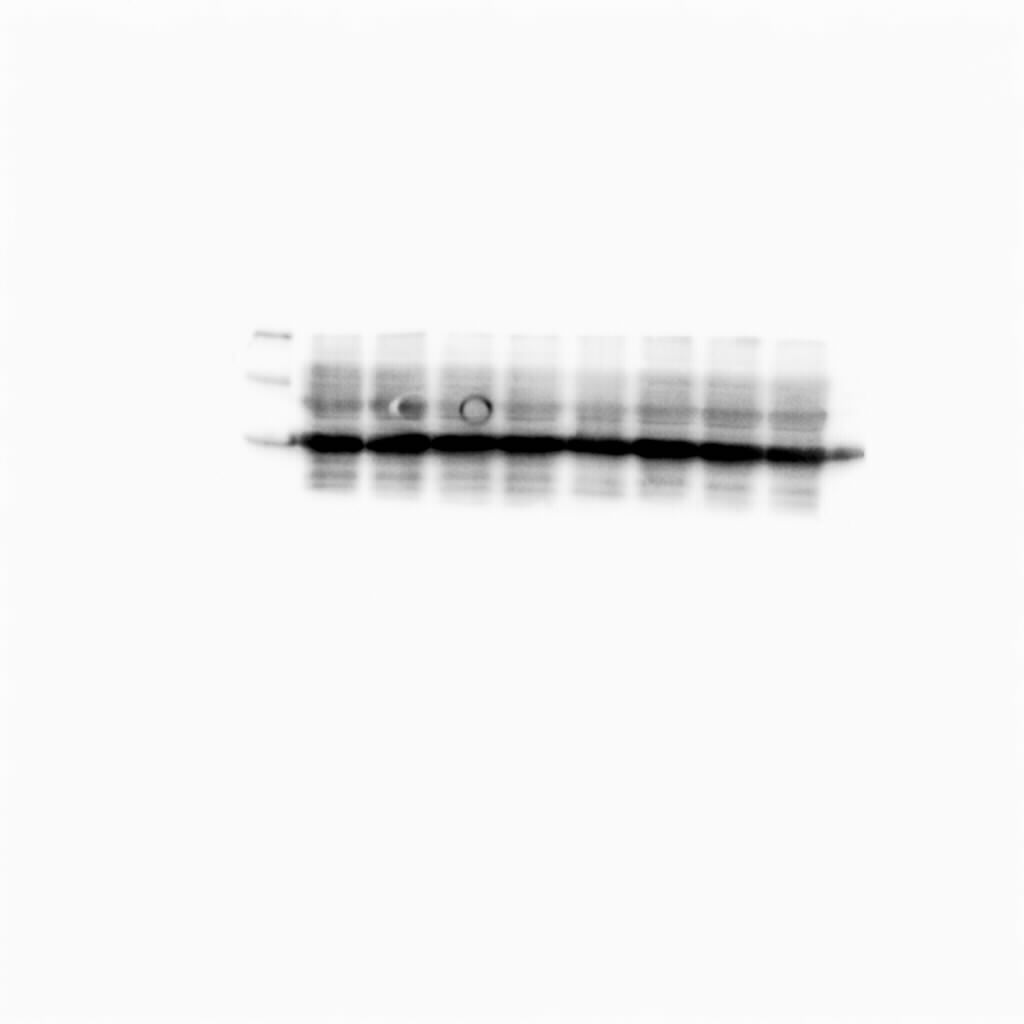


37KD

**Fig.4E**

Taok1


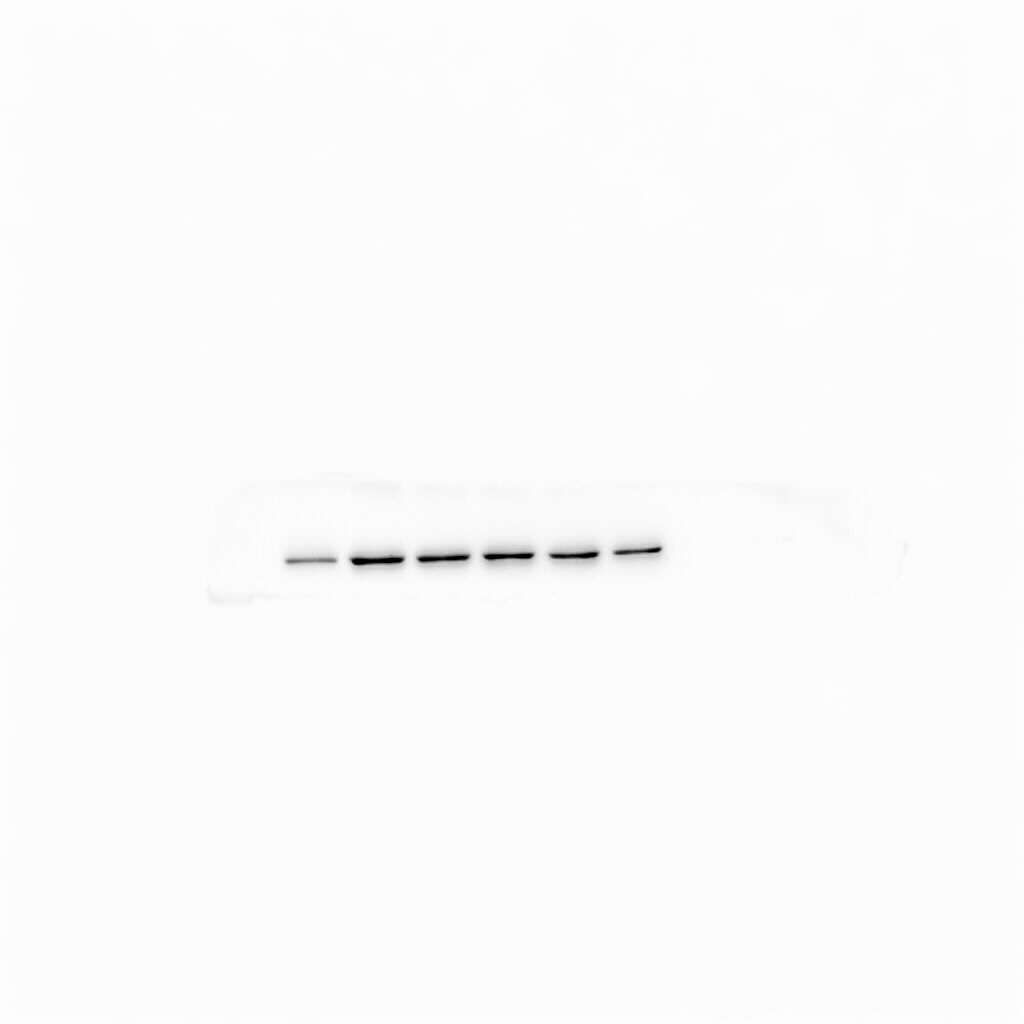


116KD

PKCα


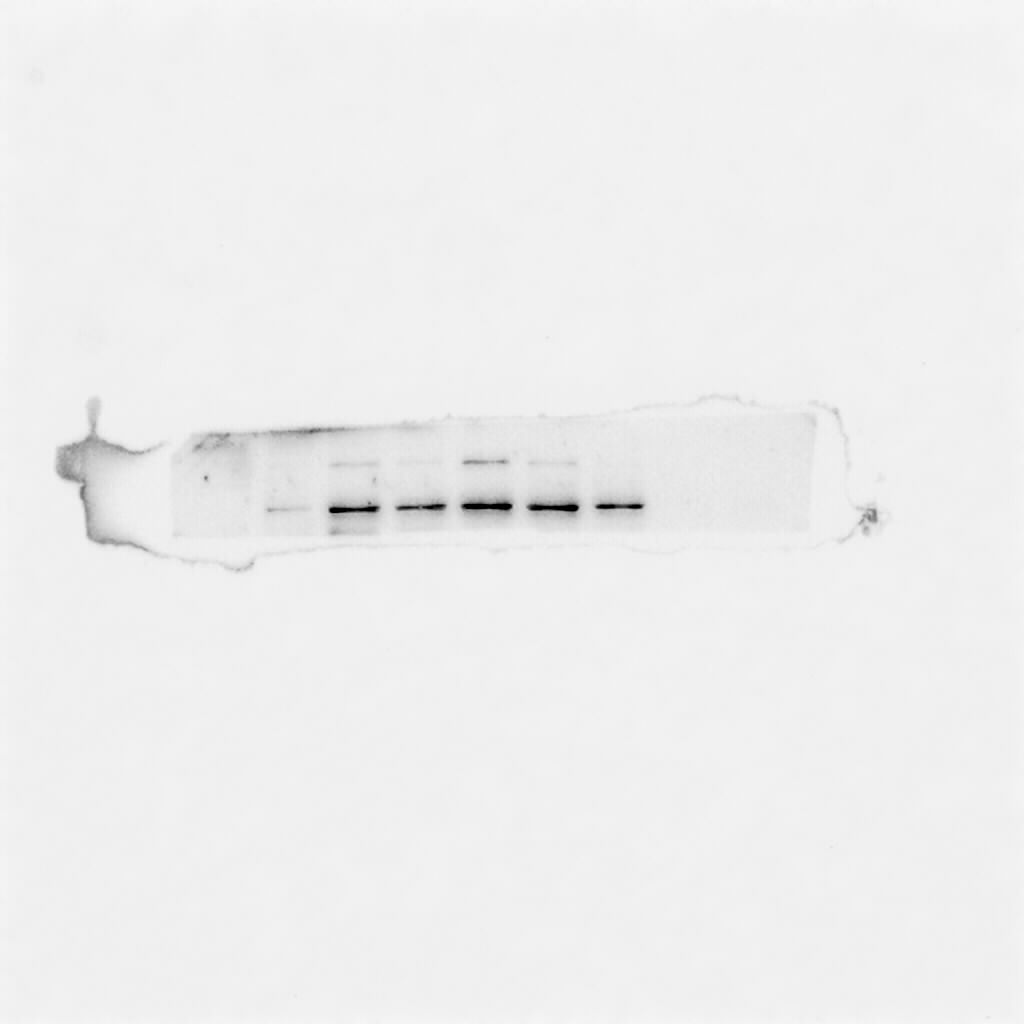


80KD

β-actin


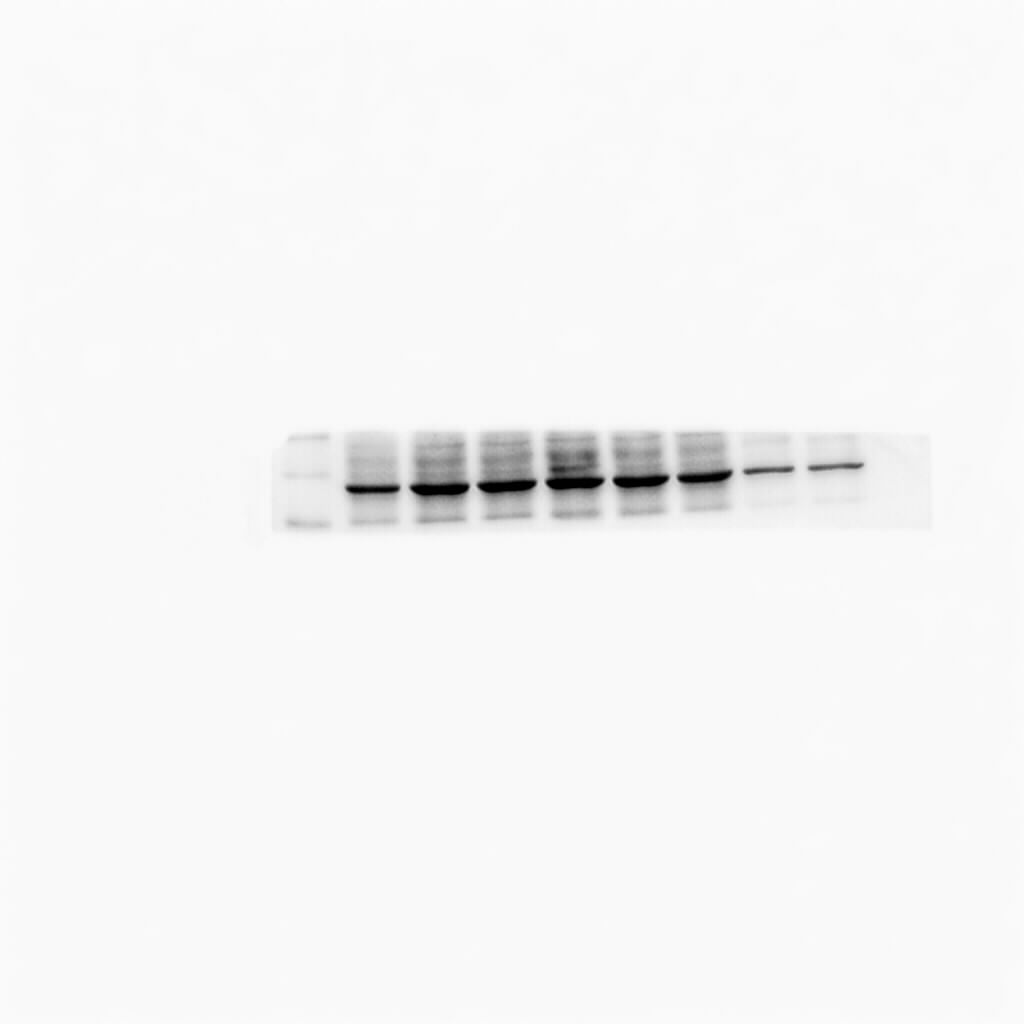


45KD

**Fig.4F**

PKCα


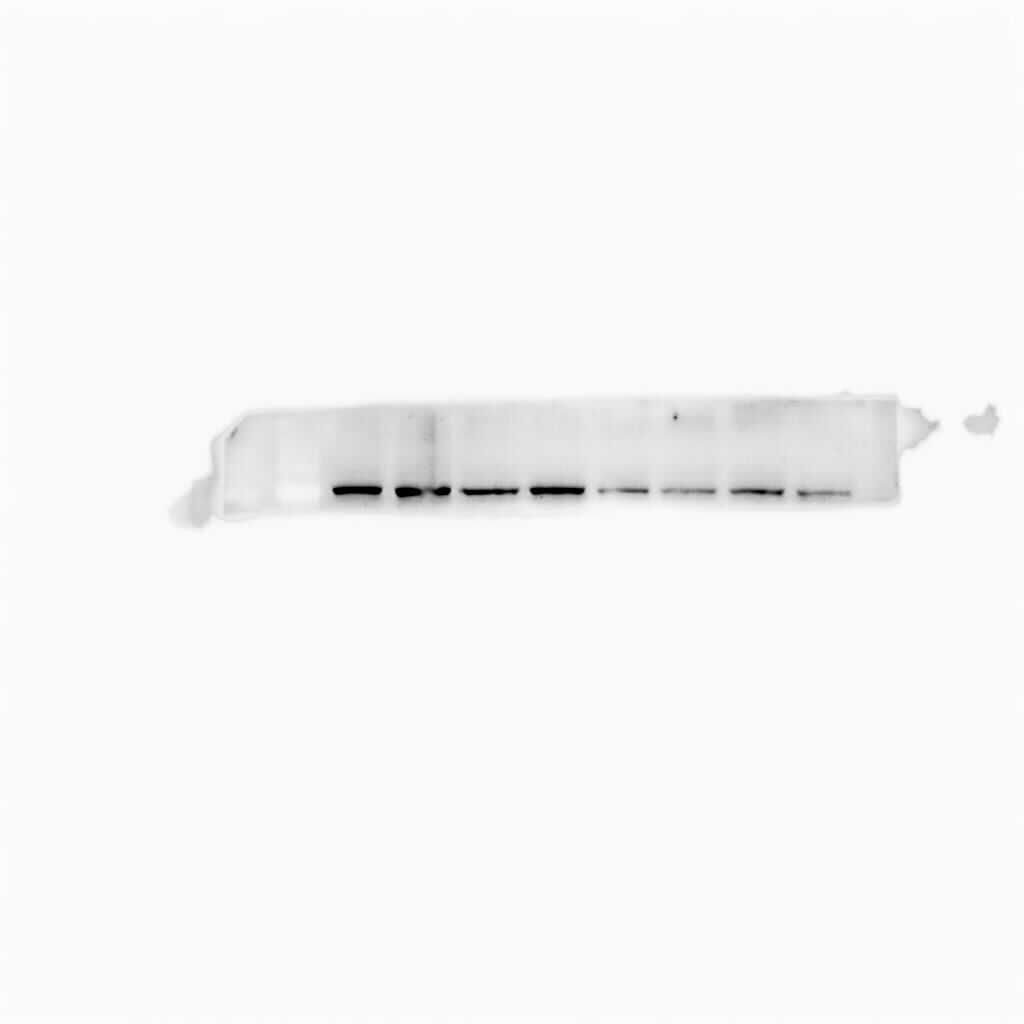


80KD

α-SMA


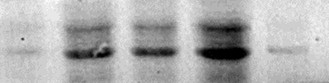


42KD

β-actin


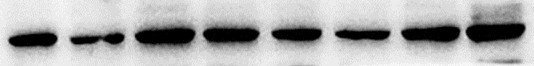


45KD

Taok1


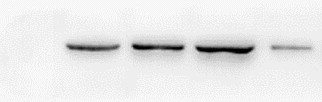


116KD

α-SMA


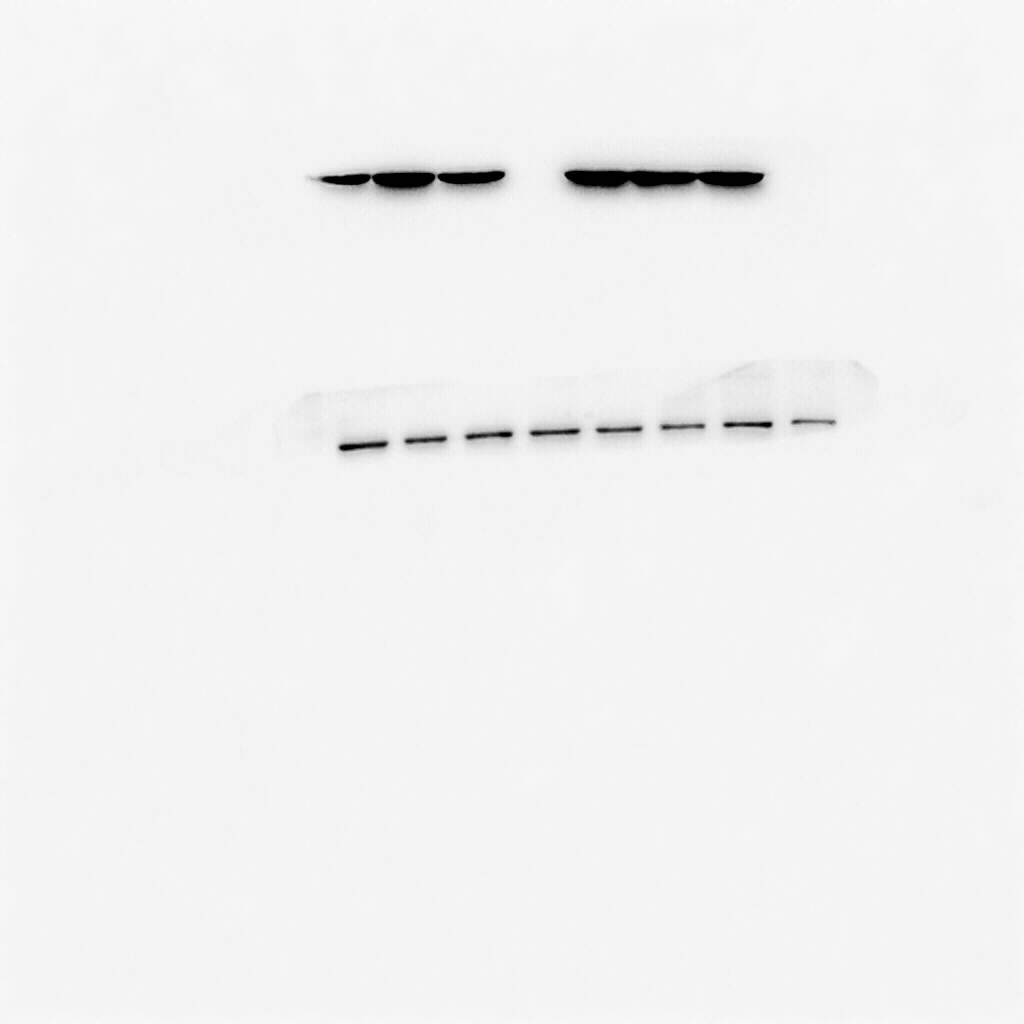


42KD

β-actin


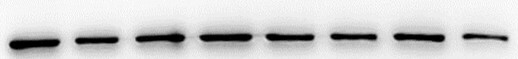


45KD

**Fig.5F**:

α-SMA


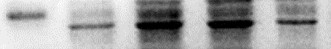


42KD


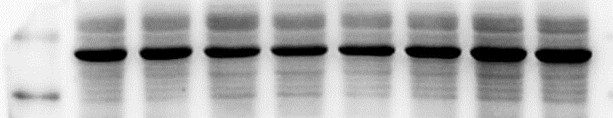
GAPDH

37KD

Col 1


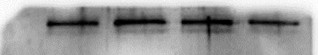


130KD

Taok1


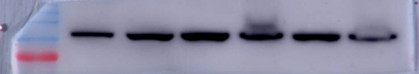


116KD

**PKCα**

**
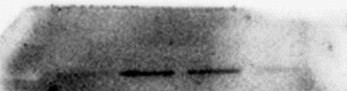
**

80KD

**Fig.6E**

**Cleaved Caspase3**

**
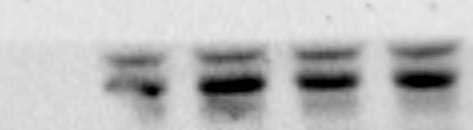
**

17/19KD

**GAPDH**

**
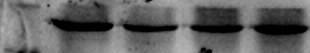
**

37KD

**Supporting Fig.2B**

HNF-4


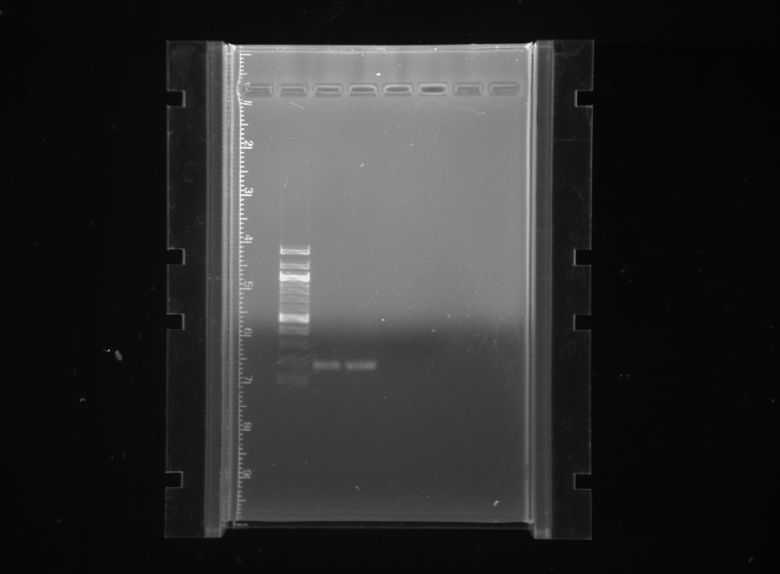


180bp

Desmin


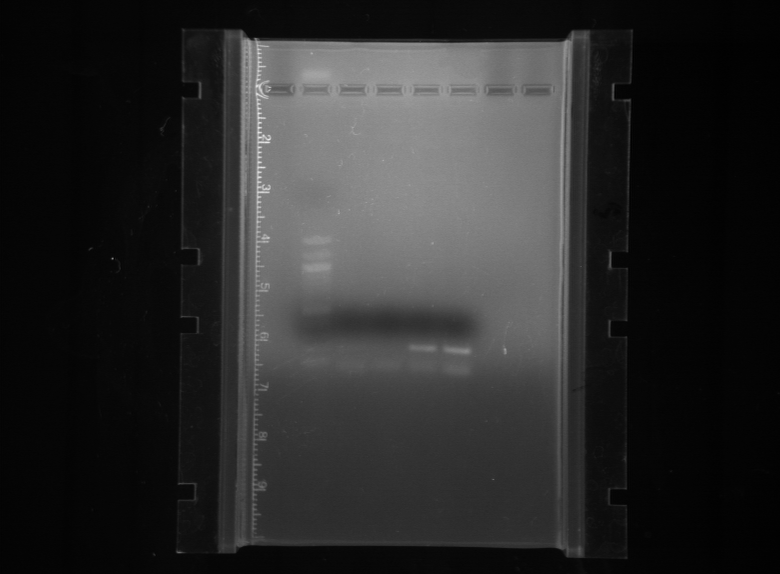


185bp

GADPH


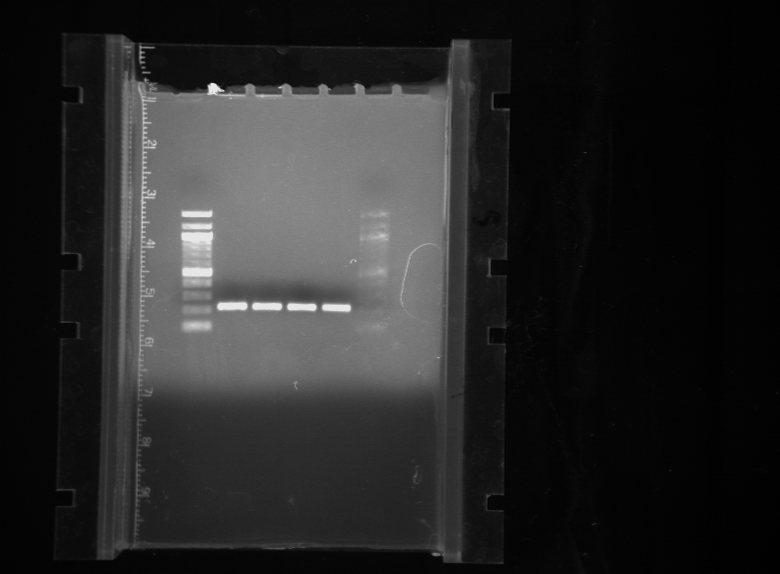


222bp

**Supporting Fig.3B**

α-SMA


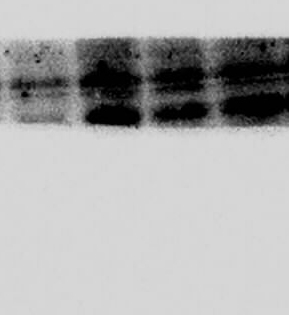


42KD

E-Cadherin


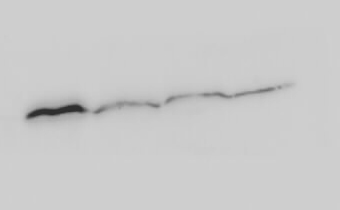


135KD

β-actin


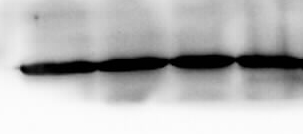


45KD

**Supporting Fig.3D**

α-SMA


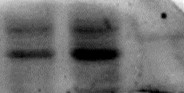


42KD

Albumin


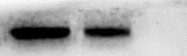


66KD

β-actin


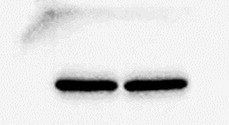


45KD

**Supporting Fig.4A**

α-SMA


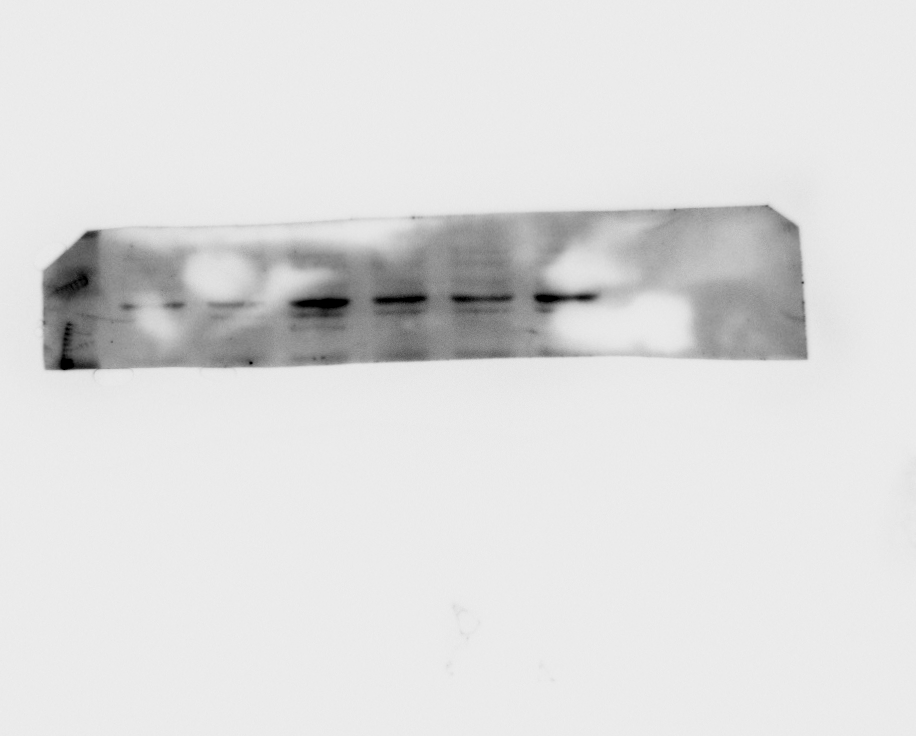


42KD

PKCα


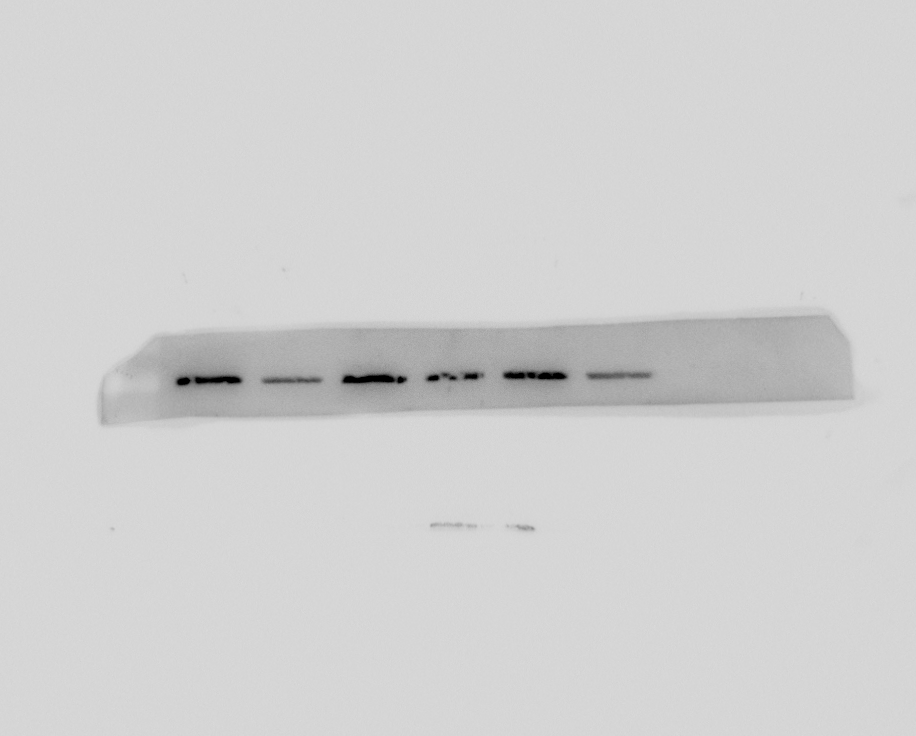


80KD

Taok1


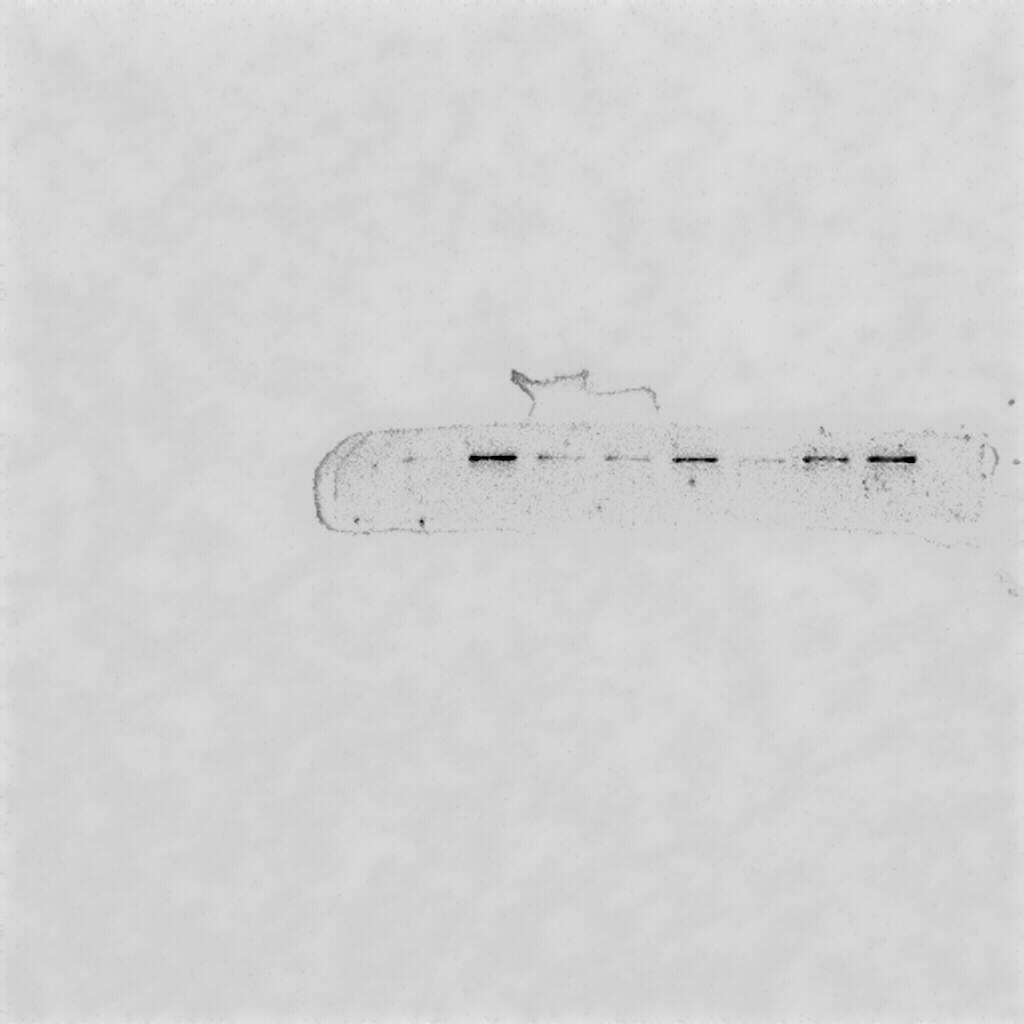


116KD

β-actin


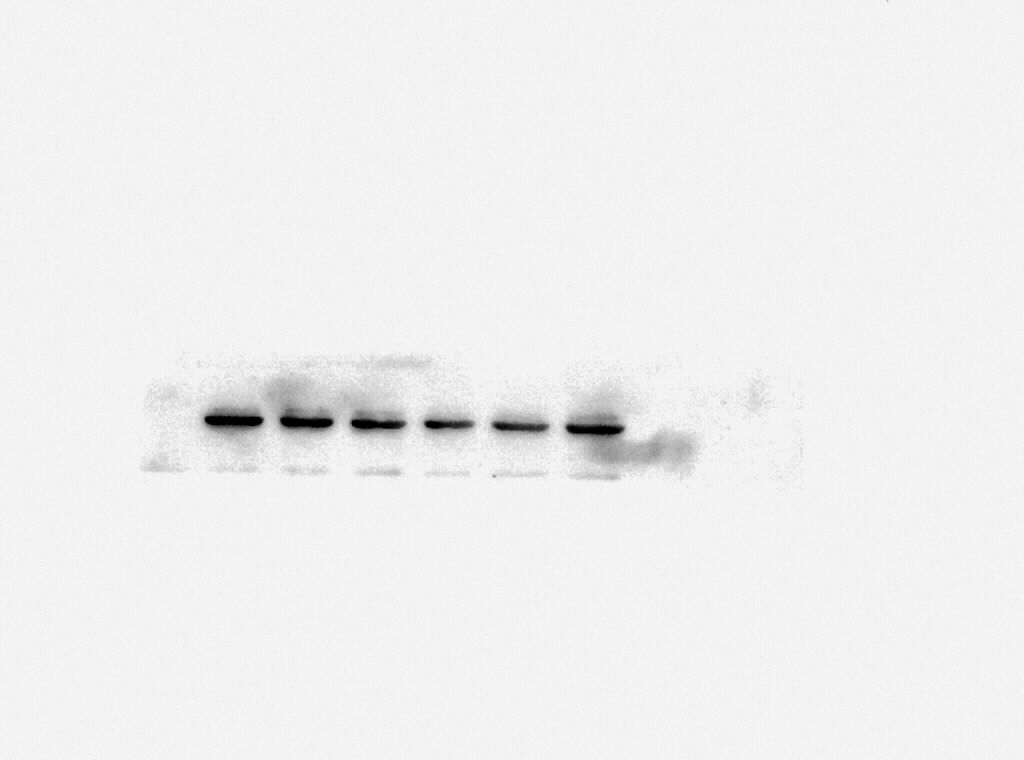


45KD

**Supporting Fig.4B**

PKCα


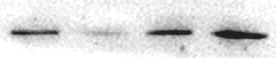


80KD

Taok1


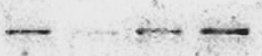


116KD

β-actin


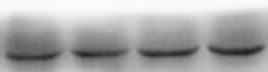


45KD

**Supporting Fig.5**

Taok1


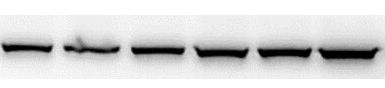


116KD

PKCα


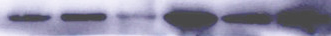


80KD

Col1


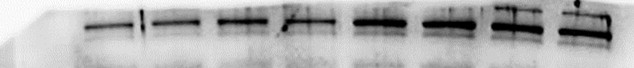


130KD

α-SMA


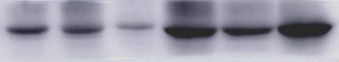


42KD

GAPDH


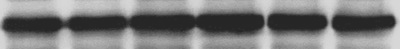


37KD
